# Blaming the Victim of Acquaintance Rape: Individual, Situational, and Sociocultural Factors

**DOI:** 10.3389/fpsyg.2018.02422

**Published:** 2019-01-21

**Authors:** Claire R. Gravelin, Monica Biernat, Caroline E. Bucher

**Affiliations:** ^1^Division of Social and Behavioral Sciences, Franklin Pierce University, Rindge, NH, United States; ^2^Department of Psychology, The University of Kansas, Lawrence, KS, United States; ^3^Department of Psychology, SUNY Geneseo, Geneseo, NY, United States

**Keywords:** acquaintance rape, blame, responsibility, sexual assault, sexual violence, victim blame

## Abstract

Victims of rape are uniquely vulnerable for being blamed for their assault relative to victims of other interpersonal crimes and thus much research has been conducted to understand why this is the case. But the study of victim blaming in acquaintance rape cases is hindered by contradictory empirical results. Early investigations in victim blaming often treated acquaintance rapes and stranger rapes as synonymous and thus much of these data are suspect for drawing conclusions particular to acquaintance rape. This paper provides a comprehensive review of the research literature on victim blame in acquaintance rape cases, highlighting inconsistencies and drawing particular attention to areas of research in need of further exploration. Specifically, we review the commonly studied individual (perceiver) factors that influence victim blaming, as well as common situational (target) factors included or manipulated within sexual assault scenarios. Our review reveals many inconsistent findings and interactions between perceiver and scenario factors. In an effort to make sense of these complex interactions and inconsistent findings, we suggest a need for more transparency in describing the scenarios used in research on victim blaming in sexual assault cases and greater empirical attention to sociocultural factors that may influence blaming tendencies.

## Introduction

For anybody whose once normal everyday life was suddenly shattered by an act of sexual violence– the trauma, the terror, can shatter you long after one horrible attack. It lingers. You don’t know where to go or who to turn to…and people are more suspicious of what you were wearing or what you were drinking, as if it’s your fault, not the fault of the person who assaulted you…We still don’t condemn sexual assault as loudly as we should. We make excuses, we look the other way…[Laws] won’t be enough unless we change the culture that allows assault to happen in the first place.

- President Barack Obama, September 2014

Sexual assault is a pressing and prevalent concern in our society with estimates that nearly 1 in 5 women in the United States will be sexually assaulted in her lifetime. Of those women who have been sexually assaulted, 41% have been assaulted by an acquaintance (Black et al., 2011). These numbers likely underestimate prevalence, as sexual assaults are one of the most under-reported crimes (Fisher et al., 2000, 2003; Rennison, 2002). In the unveiling of the “It’s On Us” campaign to end sexual assault on college campuses, President Barack Obama highlighted not only the trauma experienced by rape victims due to their assault, but also the secondary victimization many victims experience due to the negative reactions of those around them (see also Williams, 1984; Ulman, 1996). Of these negative reactions, perhaps the most harmful is the frequent tendency to blame the victim for their assault.

Unlike many other interpersonal crimes such as robberies or muggings, victims of sexual assault are particularly vulnerable to being blamed for their attack (Bieneck and Krahé, 2011; Gordon and Riger, 2011), and thus victim blaming in sexual assault cases has been the focus of many empirical investigations. However, despite the extensive amount of research performed on this topic, there is little consensus of when victim blaming will or will not occur in sexual assault cases (see Grubb and Harrower, 2008 and Grubb and Turner, 2012, for a review).

Adding to the confusion, existing reviews on victim blaming often combine the findings across various types of sexual assault (Langley et al., 1991; Pollard, 1992; Whatley, 1996; Grubb and Harrower, 2008; Grubb and Turner, 2012). For instance, Grubb and Harrower (2008) reviewed differences in victim blaming between stranger and acquaintance rape, but then combined these types of sexual assault when discussing the influence of gender and perceived similarity on victim blame. As different factors may matter for victim blaming, combining findings across sexual assault types may be problematic. The goal of this paper is to highlight what we know (and do not know) about victim blaming in acquaintance rape.

**FIGURE 1:**
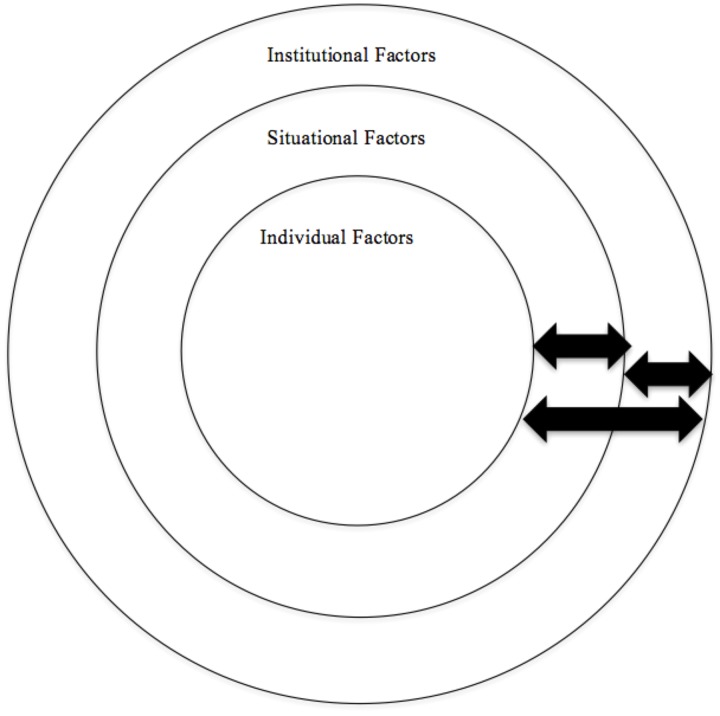
Conceptual model of the levels in which victim blame in sexual assault has been examined. Arrows serve to remind readers that these levels interact with one another and are not mutually exclusive.

The opening statement by President Obama also highlights another important and often ignored element that contributes to the continued tendency to blame victims of sexual assault – the role of cultural structures, beliefs, and practices. Research on sexual assault and victim blame typically focuses on one of two perspectives. The first considers features of the observer as they influence victim blaming tendencies, which we refer to as *individual factors*. Often discussed as the “rape perception framework,” the second perspective focuses on aspects of the victim, perpetrator, or characteristics of the assault as they influence victim blame (Pollard, 1992). We refer to these elements as *situational factors*. Neither of these perspectives, however, addresses a third critical factor affecting victim blame: societal and institutional factors. Institutional and societal level factors refer to broader cultural influences such as gender roles, media, and rhetoric surrounding sexual assault that contribute to an overall environment promoting victim blame. The current review will consider both individual-level and situational-level variables as they affect victim blaming in acquaintance rape cases but will also discuss the role of institutional and societal-level factors. Further, we consider how all three elements may influence one another (see Figure 1).

This paper is intended to provide a comprehensive review of the research literature on victim blaming in acquaintance rape and the conditions under which victim blaming is influenced by individual and situational factors. We begin by briefly defining what we mean by sexual assault, acquaintance rape, and victim blaming. We then review the research literature and propose a broader framework that includes attention to societal and institutional factors as important contributors to victim blame.

## Sexual Assault

Current conceptions of rape and sexual assault typically include penetration, whether it be genital, oral, or anal, by part of the perpetrator’s body or object through the use of force or without the victim’s consent. While not discounting the victimization of men, sexual assault is a gendered crime, with women much more likely to be victimized then men (Brownmiller, 1975; Koss et al., 1987; Koss and Harvey, 1991; Hayes et al., 2013). Indeed, compared to one in five American women, one in 71 men will be assaulted in his lifetime (Black et al., 2011). Thus, while male victimization is indeed problematic, given the highly gendered nature of this crime, the current work focuses exclusively on female victims.

Researchers investigating the prevalence and consequences of sexual assault typically distinguish among three types of sexual assault: stranger rape, date/acquaintance rape, and marital rape. Stranger rape refers to a sexual assault in which the victim and assailant have no prior relationship or acquaintance with one another. When an individual has been sexually assaulted by someone she knows – for instance a friend, classmate, or someone she has gone on a few dates with – it is classified as an acquaintance or date rape (Calhoun et al., 1976; Check and Malamuth, 1983; Estrich, 1987; Johnson and Jackson, 1988; Quackenbush, 1989), but “date rape” is also used to describe assaults that occur in established relationships (Shultz et al., 2000). Finally, sexual assault that occurs within a marriage has been deemed a legal form of rape, with the first successful marital rape conviction occurring in the United States in 1979 (Pagelow, 1988). These distinctions may not provide as much clarity as desired. For example, assault by one’s unmarried romantic partner may have more in common with marital rape than acquaintance rape; assault while on a first date may differ considerably from assault by a classmate to whom one has never spoken. The current review will focus on sexual assaults classified as acquaintance rape, and we will note distinctions between dating-related and non-dating related acquaintance rape where relevant. Gaining a greater understanding of victim blaming in acquaintance rape is particularly important given that the majority of rapes are perpetrated by someone known to the victim (Russell, 1984; Koss et al., 1988; Pfeiffer, 1990), and that acquaintance rape cases have a lower probability of conviction in the courts than those that that fit with a stranger rape script (Estrich, 1987; Larcombe, 2002).

## Blaming the Victim

Blaming the victim refers to the tendency to hold victims of negative events responsible for those outcomes (Ryan, 1971; Eigenberg and Garland, 2008). While victim blaming can occur in a variety of situations, it appears to be particularly likely in cases of sexual assault (Bieneck and Krahé, 2011). Assailants do tend to be found as more culpable for sexual assault than victims (see Grubb and Harrower, 2008), but victims are blamed as well, to a degree that varies substantially depending on features of the assault, the victim, and the perceiver.

There is currently little consensus about the predictors of victim blaming (see Grubb and Harrower, 2008; Grubb and Turner, 2012). In fact, the sexual assault literature appears to offer only one clear conclusion: Victims of stranger rape are the least likely to be blamed for their assault; victims of marital rape are much more likely to be found culpable (Ewoldt et al., 2000; Monson et al., 2000). Direct comparisons between stranger rape and acquaintance rape typically find less blame in the former case (Amir, 1971; Calhoun et al., 1976; Donnerstein and Berkowitz, 1981; L’Armand and Pepitone, 1982; Janoff-Bulman et al., 1985; Tetreault and Barnett, 1987; Muehlenhard and Hollabaugh, 1988; Bridges and McGrail, 1989; Quackenbush, 1989; Pollard, 1992; Hammock and Richardson, 1997; Sinclair and Bourne, 1998; Krahé et al., 2007; Grubb and Harrower, 2008; Bieneck and Krahé, 2011; Droogendyk and Wright, 2014; McKimmie et al., 2014; Ayala et al., 2015; Stuart et al., 2016, but see Persson et al., 2018). Further, acquaintance rape victims are blamed less than marital rape victims (Ferro et al., 2008). In short, as the victim and assailant become increasingly familiar and romantically involved, victim blame increases (Bridges, 1991; Simonson and Subich, 1999; Krahé et al., 2007; Bieneck and Krahé, 2011; Pederson and Strömwall, 2013, but see McCaul et al., 1990, and Klippenstine et al., 2007).

## Measuring Blame

The measurement of “blaming the victim” may seem straightforward, but it varies substantially in the literature. Researchers typically present participants with a scenario of a sexual assault case, then some researchers assess *blame*, others assess perceived *responsibility*, others utilize a combination of both blame and responsibility, and still others assess related constructs. *Blame* is typically defined as a value judgment of the extent to which one should be held accountable for (and perhaps suffer from) a negative event (Bradbury and Fincham, 1990; Calhoun and Townsley, 1991; Stormo et al., 1997) and is typically measured using a rating scale (e.g., How much is the victim to blame for her assault?). *Responsibility*, defined as the extent to which victims’ choices or actions contributed to their assault (Stormo et al., 1997), is typically assessed by asking participants to assign a percentage of responsibility to the involved parties. Thus, blame may be a harsher assessment than responsibility and perceivers may therefore be more comfortable in attributing responsibility than blame.

Some researchers have argued that blame and responsibility measures can be used interchangeably (Bradbury and Fincham, 1990; Calhoun and Townsley, 1991); others argue that they are distinct constructs and should be treated as such (Richardson and Campbell, 1980, 1982; Critchlow, 1985; Shaver and Drown, 1986; Richardson and Hammock, 1991). The data are inconsistent on these points. For example, Stormo et al. (1997) found their measures of responsibility and blame to be highly positively correlated (see also Krulewitz and Nash, 1979; McCaul et al., 1990), and the two measures were similarly responsive to variations of victim intoxication in sexual assault scenarios. In contrast, Richardson and Campbell (1982) found that victim *blaming* was unaffected by level of victim intoxication, but drunk victims were judged more *responsible* for assault than sober victims. Relatedly, in assessing how dating scripts influence victim culpability, Basow and Minieri (2011) found men were more likely to *blame* victims for their assault than women, while no differences emerged in their separate measure of victim *responsibility*. Of course, non-significant effects on either measure could be due to floor/ceiling effects, particularly given the high degree of correlation between the constructs (Krulewitz and Nash, 1979; McCaul et al., 1990; Stormo et al., 1997).

Victim blame has also been assessed using other related constructs, including assessments of “fault” (Jones and Aronson, 1973; Kahn et al., 1977; Ford et al., 1998) and the extent to which the victim is perceived to have “enjoyed” the experience (Simonson and Subich, 1999). Others claim that simply failing to label a rape as a rape is a form of victim blaming (Lonsway and Fitzgerald, 1994), although labeling is more commonly used as a manipulation check to ensure that participants perceive scenarios as assaults (see Maurer and Robinson, 2008). Other more general markers of victim blame that are not answered in response to a specific case include rape myth endorsement (the extent to which participants endorse “prejudicial, stereotyped, or false beliefs” about sexual assault, victims, and assailants, pp. 217; Burt, 1980) and the Attitudes Toward Rape Victims Scale (Ward, 1988). However, these assessments often reflect beliefs surrounding stranger rapes (e.g., “Rapes only occur in dark alleys,” Payne et al., 1999; Dupuis and Clay, 2013) and thus should not be used as a measure of victim blame in acquaintance or marital rape situations. We view rape myth endorsement as a potential predictor of blame in acquaintance rape, but not as an appropriate measure of victim blame itself.

This review will consider the most common conceptualizations of victim blame (blame, responsibility, and fault) that are specific to a particular victim depicted in a scenario rather than rape myth acceptance, perceived enjoyment, or labeling of an event as rape (see Table 1 for a comprehensive listing of measures used in the reviewed studies).

**Table 1:** Measurement type used to assess victim blame and situational components featured in studies included in review.

Study	Blame measure	Situational components
^∗^Abrams et al. (2003) study 1	Blame and fault	F, R
^∗^Abrams et al. (2003) study 2	Blame and fault	F, R
^∗^Abrams et al. (2003) study 3	Blame and fault	F, R
^∗^Ayala et al. (2015)	Blame and responsibility	F, R
^∗^Bell et al. (1994)	Blame and responsibility	Assailant occupation
Ben-David and Schneider (2005)	Responsibility	F, R
Bieneck and Krahé (2011)	Blame	A, F, R
^∗^Black and Gold (2008)	Responsibility	F, R, assailant occupation
Blumberg and Lester (1991)	Blame	F, R
^∗^Bongiorno et al. (2016)	Blame	F, R, RA
Branscombe et al. (1996), study 1	Blame	F, R
Branscombe et al. (1996), study 2	Blame	F, R
^∗^Calhoun et al. (1976)	Fault	SH
Cameron and Stritzke (2003)	Blame and responsibility	A, R
^∗^Casarella-Espinoza (2015)	Blame and responsibility	A, RA
^∗^Cassidy and Hurrell (1995)	Responsibility	AP, R
^∗^Coller and Resick (1987)	Blame and responsibility	A, R
^∗^D’Cruz and Kanekar (1992)	Fault	Victim willingness to go to police
Droogendyk and Wright (2014)	Blame and responsibility	AB
Dupuis and Clay (2013)	Responsibility	SH, F, R, RA
^∗^Ferrão et al. (2016)	Responsibility	AP, F, R
^∗^Frese et al. (2004)	Responsibility	A, AP, F
^∗^Ford et al. (1998)	Blame and responsibility	A, SH, F, R
George and Martinez (2002)	Blame and responsibility	F, R
Gerdes et al. (1988)	Blame	AP, F, R
^∗^Gilmartin-Zena (1983)	Responsibility	SH, R
Girard and Senn (2008) study 1	Blame	A, D
Girard and Senn (2008) study 2	Blame	A, D
^∗^Gravelin et al. (2017) study 2	Blame	A
Hammock and Richardson (1997)	Responsibility	A, F, R
^∗^Hammond et al. (2011)	Responsibility	SH, F, R
^∗^Harbottle (2015)	Responsibility	SH, F, R
^∗^Howells et al. (1984)	Blame	SH
^∗^Idsis and Edoute (2017)	Responsibility	A, SH, F, R
^∗^Janoff-Bulman et al. (1985) study 2	Blame	A
Johnson (1994)	Responsibility	SH
^∗^Johnson (1995)	Blame	SH
Johnson and Jackson (1988)	Responsibility	F, R
^∗^Johnson et al. (1995) study 2	Responsibility	F, R
Johnson et al. (1989)	Responsibility	F, R
^∗^Johnson et al. (2016)	Responsibility	A, F, R, SH
Kanekar and Nazareth (1988)	Fault	SH, physical harm and emotional disturbance after the assault
^∗^Kanekar and Seksaria (1993)	Fault	SH, F, R
Kanekar et al. (1991) study 1	Fault	Assailant and victim occupation, victim’s willingness to go to police
Kanekar et al. (1991) study 3	Fault	Victim’s marital status
Kanekar et al. (1991) study 5	Fault	F, R
Kerr and Kurtz (1977)	Blame	SH, F, R
Klippenstine et al. (2007)	Blame and responsibility	A, F, R, SH
Kopper (1996)	Blame and responsibility	F, R
Krahé et al. (2007) study 1	Blame	F, R
Krahé et al. (2007) study 2	Blame	A, F, R
^∗^Lambert and Raichle (2000) study 1	Blame and responsibility	A, R
^∗^Lambert and Raichle (2000)study 2	Blame and responsibility	A, R
Landström et al. (2016)	Blame, fault, and responsibility	A, F, R
Masser et al. (2010)	Blame and fault	F, R
^∗^Maurer and Robinson (2008)	Responsibility	F, R
^∗^McCaul et al. (1990) study 1	Blame	F
^∗^McCaul et al. (1990) study 2	Blame and responsibility	F
McKimmie et al., 2014	Blame	F, R
Miller et al. (2012)	Blame and responsibility	A, AP
Muehlenhard and MacNaughton (1988)	Responsibility	AP, SH, F, R
^∗^Munsch and Willer (2012)	Responsibility	A, R
Nario-Redmond and Branscombe (1996), study 1	Blame	F, R
Nario-Redmond and Branscombe (1996), study 2	Blame	F, R
Ong and Ward (1999)	Fault	F, R
Pederson and Strömwall (2013)	Blame	F, R
^∗^Persson et al. (2018)	Blame	F, R
Pugh (1983)	Blame	A, AP, SH, F, R
Qi et al. (2016)	Fault	A, D, F, R
Richardson and Campbell (1982)	Blame and responsibility	A, R
Root (1993)	Blame	AB
^∗^Romero-Sánchez et al. (2012) Study 1	Blame	A, F, R
^∗^Romero-Sánchez et al. (2012) Study 2	Blame	A, F, R, victim sexual attraction
Schuller et al. (2010)	Blame	F, R, Victim emotionality, gender stereotypicality
^∗^Schuller and Wall (1998)	Blame and responsibility	A, F, R
Scronce and Corcoran (1995)	Responsibility	A, F, R, attempted or completed rape
^∗^Shotland and Goodstein (1983)	Blame and responsibility	AP, F, R
^∗^Simonson and Subich (1999)	Responsibility	F, R
^∗^Sims et al. (2007)	Responsibility	A, R
Sommer et al. (2016)	Blame and responsibility	F, R, SH
^∗^Smith et al. (1976)	Responsibility	SH, F, R
^∗^Spencer (2016)	Blame	F, R, victim occupation
^∗^Stahl et al. (2010) study 1	Blame	A, AP, F, R
^∗^Stahl et al. (2010) study 2	Blame	A, AP, F, R
Starfelt et al. (2015)	Blame	A, F, R
Stormo et al. (1997)	Blame and responsibility	A, F, R
Strömwall et al. (2013)	Blame	F, R
^∗^Stuart et al. (2016)	Blame and fault	F, R, victim emotionality
^∗^Tetreault and Barnett (1987)	Responsibility	F, R
Van Den Bos and Maas (2009) study 1	Blame	AB
Varelas and Foley (1998)	Responsibility	F, R, RA
Viki and Abrams (2002)	Blame	SH
^∗^Wall and Schuller (2002)	Blame and responsibility	A, F, R
^∗^Whatley and Riggio (1992)	Responsibility	A, AP
^∗^Wiener and Vodanovich (1986)	Responsibility	F, R, assailant criminal history
Willis (1992)	Responsibility	RA
^∗^Wooten (1980)	Responsibility	R
^∗^Workman and Orr (1996)	Responsibility	AP, F, R
Wyer et al. (1985)	Responsibility	F, R
^∗^Yamawaki (2007)	Blame	F, R
^∗^Yamawaki and Tschanz (2005)	Blame	F, R
^∗^Yamawaki et al. (2007)	Blame	F, R, assailant occupation


*^∗^ = full scenarios obtained. Blame is typically measured as how much the victim is to blame for her assault (rated on a scale with endpoints such as “not at all” to “to a great extent/completely;” responsibility is typically measured as a percentage of responsibility assigned to the involved parties; fault is typically measured as how much the victim is at fault for what happened rated on a scale with endpoints such as “not at all” to “very much/extremely). Abbreviations used to depict which of the following components were present and/or manipulated in scenario(s) for each study; A, Alcohol; D, Drugs; AP, Appearance; SH, Sexual history/actions; F, Force; R, Resistance; RA, victim and/or assailant race/ethnicity; AB, Scenario details absent.*

## Methods

To identify the extant research literature, our search strategy included combinations of the keywords *rape* or *sexual assault* with *victim blame*, and limited to *date* or *acquaintance* rape in electronic databases including PsycINFO, and Proquest Dissertation and Theses published through December 2017 (inclusive). Additional articles were found by conducting forward and backward searches utilizing reference sections of retrieved articles and earlier reviews through Google Scholar. This approach yielded 137 articles, which were then assessed for fit according to our inclusion criteria. The review was restricted to studies of lay observers (e.g., studies of therapists’ tendency to victim blame and personal accounts by victims and perpetrators were excluded). In addition, only studies of victim blame in cases involving a female victim and male assailant, most often depicted via a written or visual scenario, were included. The typical study exposed participants to a vignette/scenario/description of an acquaintance rape, then assessed victim blaming. Following these exclusions, 102 empirical studies on acquaintance rape that used at least one measure of victim blame, as defined above, were located. Our goal was to identify key factors that have been considered as predictors of victim blaming in these studies, to review what has been learned about each, and to highlight inconsistencies and gaps in the literature. These factors generally fall into two categories: features of the perceiver (individual level factors) and features of the acquaintance rape itself (situational factors). We offer a narrative rather than empirical review: Meta-analysis was not appropriate to our goals given the large number of disparate predictors we considered, the often small number of cases of each type, the myriad of moderators (often unique to particular subsets of studies) that point to nuanced patterns rather than main effects.

## Results

### Individual Level Factors as Predictors of Victim Blaming

#### Gender

Given the gendered nature of sexual assault, it is unsurprising that many studies have examined how participant gender may influence evaluations of blame in sexual assault (see Grubb and Harrower, 2008 for a review). There are two contradictory hypotheses one might have about how gender affects victim blaming. On the one hand, because rape is mainly a concern of women, they might be expected to blame less as a function of ingroup solidarity. On the other hand, “just world” ideology (Lerner, 1970, 1980; Hafer, 2000) might suggest they might blame more: Precisely because of the greater threat that sexual assault poses to women, victim blaming may help women distance themselves from the reality that they could be victimized themselves.

Many studies have found that women are less likely to blame victims of acquaintance rape than men (Basow and Minieri, 2011, although gender differences only emerged in their assessment of victim *blame*, and not in their separate measure of victim *responsibility*; Calhoun et al., 1976; Selby et al., 1977; Gerdes et al., 1988; Johnson and Jackson, 1988; Kanekar and Nazareth, 1988; Johnson et al., 1989; Bell et al., 1994; Schuller and Wall, 1998; Varelas and Foley, 1998; Lambert and Raichle, 2000; Geiger et al., 2004; Klippenstine et al., 2007; Krahé et al., 2007; Yamawaki et al., 2007; Black and Gold, 2008; Hammond et al., 2011; Casarella-Espinoza, 2015; Ferrão et al., 2016). A number of other studies, however, have produced null effects of gender on victim blaming (Gilmartin-Zena, 1983; Howells et al., 1984; Krahé, 1988; McCaul et al., 1990; Kanekar et al., 1991; Kanekar and Seksaria, 1993; Branscombe et al., 1996; Nario-Redmond and Branscombe, 1996; Hammock and Richardson, 1997; Abrams et al., 2003; Frese et al., 2004; Girard and Senn, 2008; Bieneck and Krahé, 2011; Romero-Sánchez et al., 2012; Loughnan et al., 2013; Pederson and Strömwall, 2013; Bongiorno et al., 2016; Landström et al., 2016; Qi et al., 2016; Persson et al., 2018; although these studies assessed victim culpability for being “sexually touched” at a bar and thus it is unclear if a rape has occured; Scronce and Corcoran, 1995; Stormo et al., 1997; Sims et al., 2007; Strömwall et al., 2013). No studies have found that women engaged in greater victim blaming than men. Thus, the just world prediction currently does not receive support.

A meta-analysis conducted by Whatley (1996) on victim blaming failed to find significant moderation of blame by participant gender. It is problematic to draw any conclusions from this meta-analysis, however, as it combined studies of acquaintance rape with stranger rape. Meta-analyses on rape myth endorsement do indicate men are more accepting of rape myths than women (Anderson et al., 1997; Suarez and Gadalla, 2010). However, as previously noted, rape myths are a problematic marker of victim blaming in acquaintance rape since rape myths more closely reflect stranger rape situations.

We suspect that the inconsistent findings regarding the role of participant gender on blame are likely due to varying components of the scenarios used in victim blaming studies. For instance, Bell et al. (1994) failed to find gender differences, but the scenarios used were brief and vague (only two sentences long). Hammond et al. (2011) exposed participants to a lengthy scenario, several paragraphs long and rich in detail including background information about both the victim and assailant and information about behavior prior to the assault (heavy drinking and flirting). In this study, women were found to blame the victim significantly less than men.

#### Race/Ethnicity/Nationality

Very little research has examined the effect of participant race or ethnicity on victim blaming in acquaintance (or stranger) rape. Of those studies that have done so, the findings are inconsistent. Bell et al. (1994) study of undergraduates’ reactions to a “typical” date rape scenario (victim assaulted after a date), found no effect of participant race (African American, Asian, and Caucasian participants) on victim blaming. Casarella-Espinoza (2015), however, found greater victim blaming among Hispanic participants compared to their Caucasian counterparts. While both of these studies examined blame within a scenario that was likely interpreted as involving a White victim and White assailant, Varelas and Foley (1998) examined how participant race might interact with race of perpetrator or victim. White and Black participants were randomly assigned to read an acquaintance rape scenario that depicted a Black or White female victim and a Black or White male assailant. In general, White participants were less likely to blame victims than Black participants. This main effect, however, was qualified by a significant three-way interaction with victim and assailant race: White participants blamed victims the least when the victim was White and the assailant was Black, while Black participants blamed victims the most when the victim was Black and the assailant was White.

The discrepancies between these two studies could be due to the differing scenarios used (assault after a date versus assault after accepting a ride home from a customer), or to the differing ways in which blame was evaluated (blame versus responsibility), or to the important moderating feature of assailant and victim ethnicity. In any case, related literature provides some support for the argument that, at least in the United States, minority group members may blame sexual assault victims more than ethnic majority members (Caucasians; cf. Feild, 1978; see also Lonsway and Fitzgerald, 1994). Several studies assessing general attitudes toward rape victims and endorsement of rape myths have found less favorable reactions and greater endorsement of rape myths among African-American samples (Williams and Holmes, 1981; Giacopassi and Dull, 1986; Dull and Giacopassi, 1987), Asian-American samples (Mori et al., 1995), and Hispanic-American samples (Fischer, 1987; Jimenez, 2002; Jimenez and Abreu, 2003) in comparison to their Caucasian counterparts (see Suarez and Gadalla, 2010, for a review). Future research should continue to explore the effect of participant race and ethnicity on victim blaming in acquaintance rape cases, especially in combination with race/ethnicity of victim and assailant.

Relatively few studies have compared participants from differing racial/ethnic groups outside of a North American context. Exceptions include Pederson and Strömwall (2013), who compared British and Swedish non-student participants’ victim blaming in an acquaintance rape scenario and found no differences. Yamawaki and Tschanz (2005) compared Japanese and American undergraduate students and found higher victim blaming by Japanese than American students (this was true for stranger, acquaintance, and marital rape depictions). In an Australian sample, Bongiorno et al. (2016) found that a non-resisting victim was seen as more blameworthy when her perpetrator was characterized as being culturally similar (Western) to the participant, but cultural similarity had no effect when the victim physically resisted the assault.

#### Rape Myth Endorsement (RME)

As previously stated, some researchers have used RME as an indicator of victim blame. This is problematic because rape myth scales focus on stranger rape and assesses beliefs about rape at a general rather than specific level. Nonetheless, RME may matter for assessing blame in particular acquaintance rape cases. Those high in RME tend to believe that only stranger rape is “real rape.” Given that acquaintance rapes deviate from stranger rape both in recognition as rape as well as perceived severity (e.g., L’Armand and Pepitone, 1982; Tetreault and Barnett, 1987; Gerdes et al., 1988; Bridges, 1991), endorsement of rape myths may predict even greater victim blaming in acquaintance rape as these do not fit typical conceptualizations of a “real” rape.

Research clearly supports a positive relationship between endorsement of rape myths and victim blaming in acquaintance rape cases (Lonsway and Fitzgerald, 1994; Stormo et al., 1997; Schuller and Wall, 1998; Varelas and Foley, 1998; Frese et al., 2004; Hayes-Smith and Levett, 2010; Masser et al., 2010; Basow and Minieri, 2011; Hammond et al., 2011; Romero-Sánchez et al., 2012; McKimmie et al., 2014; Starfelt et al., 2015; Qi et al., 2016; Persson et al., 2018). Additionally, the relationship between rape myth endorsement and greater victim blame tends to be strongest among men (Lonsway and Fitzgerald, 1994; Hayes-Smith and Levett, 2010; Hammond et al., 2011). Using a related construct, the Perceived Causes of Rape scale (Cowan and Quinton, 1997), Krahé et al. (2007) found that some subscales of this instrument showed the strongest positive associations with victim blaming: beliefs that rape is due to female teasing and to male pathology, and that men lack control over their sexual urges.

#### Gender Role Attitudes and Identity

Rape Myth Endorsement is significantly correlated with restrictive beliefs about women’s roles and rights (see Suarez and Gadalla, 2010). Studies of victim blame in acquaintance rape have also documented a positive relationship between blame and endorsement of traditional gender roles (Howells et al., 1984; Stormo et al., 1997; Simonson and Subich, 1999; Yamawaki and Tschanz, 2005; Sims et al., 2007, but see Hammond et al., 2011). In fact, Simonson and Subich (1999) found that after controlling for gender role endorsement, their finding that men blamed the victim more than women was eliminated; gender role attitudes may be a stronger predictor of blame than participant gender. In one study that manipulated the gender traditionality of the date that preceded an acquaintance rape, victim responsibility and perceived justifiability of the assault were highest in the traditional case (when the man exclusively paid for an expensive date) compared to other scenarios (shared payment, inexpensive date; Basow and Minieri, 2011).

Others have examined the effects of hostile and benevolent sexism (Glick and Fiske, 2001) on victim blame. Several researchers have documented a positive relationship between benevolent sexism and victim blame (Abrams et al., 2003; Masser et al., 2010, although this effect was only present among victims perceived to violate victim and gender stereotypes; Viki and Abrams, 2002; Yamawaki et al., 2007; Pederson and Strömwall, 2013; Persson et al., 2018). The relationship between hostile sexism and victim blaming, however, is more complex. For instance, Pederson and Strömwall (2013) found no relationship, while others have found hostile sexism (Masser et al., 2010; Persson et al., 2018) to predict greater victim blame (Yamawaki et al., 2007; Masser et al., 2010; Persson et al., 2018).

Both benevolent and hostile sexism reflect concerns about maintaining an unequal power differential between men and women: Benevolently sexist attitudes suggest women are lower in status and in need of men’s protection, and hostile sexist attitudes suggest that women are trying to usurp men’s greater power. Feminist perspectives point to power as a motivation for committing sexual assault (Brownmiller, 1975; Burt, 1980; Lonsway and Fitzgerald, 1994; Ward, 1995) and the effects of these attitudes on victim blame may be construed as legitimizing the current power hierarchy and maintaining gender differentiation. Indeed, research on “precarious manhood” demonstrates that masculinity, unlike femininity, is tenuous and requires continual social validation and defense (Vandello et al., 2008; Vandello and Bosson, 2013). The importance of power dynamics for victim blaming points to the need to consider the societal power structure. For example, victim blaming may increase in settings in which men perceive power threats by women (e.g., in patriarchal versus egalitarian settings).

Gender identification and threats to masculinity/femininity have also been shown to influence victim blaming. In one study, for example, participants received bogus feedback on a “gender identity survey” which either confirmed or threatened their gender identity and then were asked to evaluate a case of acquaintance rape (Munsch and Willer, 2012). Men whose masculinity was threatened blamed the victim more than those whose masculinity was confirmed. Conversely, women whose femininity was threatened blamed the victim less than non-threatened women. Thus, threats to one’s gender identity may heighten the dominant response among men and women resulting in greater blame among men and lesser blame among women, especially among men who derive a large component of their self-concept from their masculinity.

#### Political Attitudes

People who endorse more politically conservative views are also more likely to blame victims of sexual assault (see Anderson et al., 1997 for a review). For example, Lambert and Raichle (2000) found this relationship using three distinct measures of conservatism [self-rating of conservatism, social dominance orientation (Sidanius et al., 1996) and Protestant work ethic beliefs (Katz and Hass, 1988)]. Across all three measures, the more politically conservative the participants were, the more they blamed the victim.

#### Belief in a Just World (BJW)

It is commonly thought that individuals blame victims in order to restore their belief that “good things happen to good people, and bad things happen to bad people” (Lerner, 1970, 1980; Hafer, 2000). The theory of BJW describes victim blaming as a bias that enables people to maintain their beliefs in a predictable and stable environment (Lerner, 1970, 1980; Rubin and Peplau, 1973; Lerner and Miller, 1978) and therefore victim blame should increase to the extent that situations threaten BJW (Hafer, 2000).

But there is little empirical support for the association between just world beliefs and victim blaming in acquaintance rape cases (see Lambert and Raichle, 2000; Hammond et al., 2011; Pederson and Strömwall, 2013; Strömwall et al., 2013; for an exception, see Landström et al., 2016). One study did find that endorsement of BJW predicted blame for victims of sexual assault, but this was only the case among participants placed in a rationalistic mindset (defined as deliberate and effortful processing; Van Den Bos and Maas, 2009). This finding points to a potential reason behind the relative lack of effects of BJW beliefs on victim blaming: Researchers who stress that participants respond with their first, “gut-level,” reaction may be bypassing more effortful thought which allows the effect of BJW to influence victim evaluations. It is also possible that BJW more strongly impacts assessments of stranger rape, with high BJW endorsers more likely to blame the victim (Kleinke and Meyer, 1990; Strömwall et al., 2013). In their assessment of belief in a just world on victim assessment across varying relationship types, Strömwall et al. (2013) found BJW to be meaningfully related only to assessments of stranger rape. Specifically, women high in belief in a just world were significantly more likely to blame the victim than women low in belief in a just world, while BJW had no impact on male evaluations of victims.

#### Perceived Similarity and Prior Victimization

The degree to which individuals identify with a victim, either at a superficial level such as similar occupation or attitudes, or at a personal level due to their own experience with victimization, may play a role in evaluations of victim culpability. Perceived similarity to a victim may increase empathy for her experience, resulting in lesser blame (Krebs, 1975). However, it is also possible that greater feelings of similarity, particularly among female observers, heighten feelings of personal threat and distancing through victim blaming. Unfortunately, we found only three studies which assess the role of similarity on victim blaming in acquaintance rape; their findings are inconsistent. Johnson (1995) found no effect of similarity (measured as the extent to which participants felt the victim was “like them”) on victim blaming, while Bell et al. (1994) and Harbottle (2015) found that the more similar participants felt to the victim (measured with “how similar do you feel to the woman in this scenario?”), the less they blamed her. In studies of stranger rape, there is also no clear indication of the role that similarity plays on victim evaluation (see Fulero and DeLara, 1976; Kahn et al., 1977; Thornton, 1984).

Participants’ prior sexual victimization may also serve as an important contributor to perceived similarity to victims. There is little evidence that prior victimization influences victim blame in acquaintance rape (Coller and Resick, 1987; Bieneck and Krahé, 2011; Harbottle, 2015; Gravelin et al., 2017). Unfortunately, the one study located which did mention a difference in blame assessments between victims and non-victims failed to disclose *how* they differed (Johnson, 1995).

### Summary of the Effects of Individual Factors on Victim Blame

Myriad individual factors have been examined in studies of victim blame in acquaintance rape, but only a few of these factors have produced consistent findings. Developing a demographic profile of what “type” of participant is most likely to blame victims is limited by a lack of research examining racial/ethnic and national differences, and a focus on college-aged students in Western settings. It does seem to be the case that men endorse rape myths more than women (Lonsway and Fitzgerald, 1994; Hayes-Smith and Levett, 2010; Suarez and Gadalla, 2010; Hammond et al., 2011), but the effect of gender on blame in specific cases of acquaintance rape is less clear-cut.

Furthermore, any effects of participant gender may be due more to endorsement of gender roles and identification with one’s gender identity than participant gender itself. Those who endorse traditional gender roles tend to blame victims more, and controlling for gender role endorsement may eliminate effects of gender (Simonson and Subich, 1999). Further, threats to one’s masculinity/femininity appear to heighten the prototypical gendered response to victim blaming; men blame victims more and women blame victims less when their gender identity is threatened. Also interacting with gender is RME: Men generally endorse rape myths more than women, and individuals who endorse rape myths engage in more victim blaming. Similarly, men tend to be more politically conservative than women (Pratto et al., 1997; Eagly et al., 2004), and political conservatism predicts victim blaming (Anderson et al., 1997, though only one study has examined this relationship in acquaintance rape scenarios; Lambert and Raichle, 2000).

Some findings also hint at the role of social power in evaluations of victim blame. Both benevolent sexism and the power relations subcomponent of the hostile sexism scale are concerned with maintaining an unequal power differential between men and women. Endorsement of these attitudes predicts greater victim blaming (Viki and Abrams, 2002; Abrams et al., 2003; Yamawaki et al., 2007; Pederson and Strömwall, 2013). Though not described above, one set of studies in which participants’ feelings of power and powerlessness were manipulated suggest that powerless men blame victims less than men in a control condition and powerful women tend to blame the victim more than those in a control condition (Gravelin et al., 2017). These findings suggest a need to further consider patriarchal power differentials, a topic we discuss later in the paper.

Despite its direct relevance to issues of victim blame, few studies have examined the association between BJW and victim blaming, and little supportive evidence has been found. Relatedly, examinations of the effects of perceived similarity to the victim have found some, though limited evidence that those who feel more similar to the victim blame her less for her assault (Bell et al., 1994; Harbottle, 2015). No research establishes a link between prior victimization and subsequent blame of a victim in an acquaintance rape scenario.

As discussed when we reviewed each factor, some of the inconsistencies in the literature may be due to the large variety of scenarios that have been used in the victim blaming literature. Much about victim blaming may have to do with the specifics of the scenario itself, as we know, for example, from the finding that blame is greater in acquaintance rape than stranger rape cases overall. And rather than main effects of demographic and attitudinal factors, these factors may differentially matter depending on the specifics of the scenarios or cases participants are asked to consider. In the section that follows we will detail the different aspects of acquaintance rape vignettes that have been implemented or manipulated in the set of studies under review and will highlight instances in which these situational factors interact with individual factors to influence victim blame.

### Situation Level Factors as Predictors of Victim Blaming

Studies of victim blaming in acquaintance rape cases typically assess participant responses to a provided vignette. These vignettes typically consist of a third-person written account of a sexual assault (but see Janoff-Bulman et al., 1985; Tetreault and Barnett, 1987; Willis, 1992; Dupuis and Clay, 2013), in which various components of the case, the victim, and/or the assailant are manipulated. Below we review the most common elements included and/or manipulated in acquaintance rape scenarios and corresponding findings for these elements. However, of the 102 studies evaluated, only 50 included the full scenarios in their published accounts. After attempting to contact all of the authors with missing vignettes, we were able to obtain the full scenarios of an additional 2 studies, resulting in a total of 52 full scenarios for evaluation. The remaining studies were coded and evaluated based on the available information described by the authors (see Table 1 for a comprehensive list of components found within scenarios).

#### Presence of Drugs/Alcohol

Drugs and alcohol are common elements of acquaintance rape cases, particularly those that occur on college campuses (Abbey et al., 1996; Benson et al., 2007; Kilpatrick et al., 2007; Krebs et al., 2009). Much research has established a link between alcohol consumption and sexually aggressive behavior (Muehlenhard and Linton, 1987; Koss and Gaines, 1993; Ullman et al., 1999; Locke and Mahalik, 2005). As seen in Table 1, 34 of the 102 acquaintance rape vignettes in the identified literature mention alcohol. This does not include not the widely used Abrams et al. (2003) scenario, which does not explicitly mention alcohol but implies it by describing the victim as flirting and dancing all night at a party, then inviting the perpetrator home for coffee. Only sixteen of these studies experimentally manipulated the presence/absence of alcohol or varying degrees of intoxication; the remaining vignettes simply indicated alcohol use as a stable characteristic in the scenario.

Eleven of the sixteen studies that manipulated intoxication level found that intoxicated victims were blamed more for an acquaintance rape than sober victims (Richardson and Campbell, 1982; Stormo et al., 1997; Wall and Schuller, 2002; Cameron and Stritzke, 2003; Krahé et al., 2007; Sims et al., 2007; Bieneck and Krahé, 2011; Romero-Sánchez et al., 2012; Landström et al., 2016; Qi et al., 2016), and another found a linear increase in victim blame with level of victim intoxication (Stormo et al., 1997). The opposite effect of intoxication emerged for evaluations of the perpetrator: the more drunk the perpetrator, the more participants excused his behavior (see also Richardson and Campbell, 1982; Cameron and Stritzke, 2003; Johnson et al., 2016; Qi et al., 2016). Using adapted versions of the Stormo et al. (1997) vignettes, Girard and Senn (2008) found that only when the victim was depicted as having received drinks that were stronger than those of her date without her knowledge was she seen as less responsible. Research by Scronce and Corcoran (1995) suggests that women may be more critical of intoxicated victims; female participants found victims of completed or attempted stranger or acquaintance rape as more responsible for their assault if they had been drinking. Examining the combined effects of assailant and victim intoxication further complicates assessments of culpability; research by Klippenstine et al. (2007) found the typical gender effect on victim blame was nullified when both parties were depicted as intoxicated. Further, women blamed victims more than men when the victim was depicted as sober and the assailant as intoxicated.

These studies indicate both that alcohol use is a common feature of acquaintance rape scenarios used in research and that it matters for victim blaming. We suspect that many of the studies for which we could not identify precise scenario content also included alcohol use, a reflection of the common image of acquaintance rape. A more comprehensive understanding of alcohol’s role will require that researchers provide complete details about their case scenarios and that this feature be systematically manipulated.

In addition to alcohol use, there is increased societal concern about the use of “date rape drugs” in sexual assaults. Despite this concern, only one study has investigated the effect of date rape drugs on victim blame in acquaintance rape (Girard and Senn, 2008), and these researchers found that the voluntariness of drug use was crucial: Only when the victim voluntarily consumed gamma-hydoxybutric acid (GHB), an intoxicating sedative, prior to an assault was she seen as more blameworthy than a sober victim. Interestingly, a victim who was slipped GHB unknowingly was *not* seen as less blameworthy than a sober victim assaulted by a sober perpetrator. Marijuana use was examined in one study, with results mirroring the common trend found with alcohol consumption. Victims intoxicated by marijuana or alcohol are perceived as more blameworthy for their assault, while perpetrators intoxicated by the same substances are perceived as less blameworthy (Qi et al., 2016).

#### Appearance and Sexual History

Factors related to a victim’s appearance (physical attractiveness, style of dress) and sexual history (sexual orientation, previous sexual partners) are often described or manipulated in research using acquaintance rape scenarios, though less so than in studies of stranger rape. As can be seen in Table 1, 32 studies included some mention of victim attractiveness, appearance, or sexual history, and 15 of these studies manipulated some component of this information. Understanding how these elements may influence victim blaming tendencies is important given their ties to many rape myths (e.g., “It is usually only women that are dressed suggestively that are raped,” “A lot of women lead men on and then cry rape”; Payne et al., 1999).

A common misconception is that the act of rape is based on sexual desire and therefore attractive victims “ask for it” by being desirable. In domains outside of sexual assault, however, researchers often find that attractive individuals are seen as more responsible for good outcomes than for bad, while unattractive individuals are seen as more responsible for bad outcomes (Dion et al., 1972; Seligman et al., 1974; Stephan and Tully, 1977). Using a manipulation of victim attractiveness through accompanying photographs, two studies on acquaintance rape supported this pattern (Gerdes et al., 1988; Ferrão et al., 2016), though Gerdes et al. (1988) found no effect of assailant attractiveness. However, in both of these studies, the scenarios used were markedly different from the traditional account of an acquaintance rape: in one, the victim was accosted in a dark stairwell (Gerdes et al., 1988) and in the other, the victim was a married mother of two children (Ferrão et al., 2016). These features make it difficult to draw definitive conclusions about the effect of victim attractiveness on victim blame. While it is unclear whether the assault was a stranger or acquaintance rape, research conducted by Kanekar and Nazareth (1988) also failed to find a main effect of victim attractiveness on attribution of victim fault for their assault. Attractiveness was found to produce more blame only among female participants when the victim was also described as physically unharmed from the assault and not emotionally disturbed as a result of the rape. Further, female participants also judged unattractive victims as more blameworthy compared to their male counterparts when the victim was also described as unharmed and emotionally disturbed from the assault.

A more frequently studied aspect of appearance is the clothing “revealingness” or provocativeness of the victim. A scenario used by Muehlenhard and MacNaughton (1988); see also Workman and Orr (1996) described the victim as either dressing and acting provocatively (low-cut blouse, mini skirt, heels, kissing the assailant) or conservatively (high necked blouse, pleated woolen skirt, keeping a distance). Perhaps unsurprisingly, the more revealing or suggestively dressed the victim, the more the victim was blamed for her assault (Gilmartin-Zena, 1983; Cassidy and Hurrell, 1995, although these results are confounded as the conservatively dressed victim was attacked by a stranger; Muehlenhard and MacNaughton, 1988; Kanekar and Seksaria, 1993; Workman and Orr, 1996; Loughnan et al., 2013). Similarly, a victim described as wearing a body-hugging dress and high heels, compared to a more conservatively dressed victim, was viewed as having “led the perpetrator on,” leading to less perpetrator blame (but no effect on victim responsibility; Johnson et al., 2016). Using similar scenarios, Whatley and Riggio (1992) found a significant interaction between clothing style and participant gender: Men, but not women, attributed less responsibility to a conservatively dressed victim than a provocatively dressed victim.

Two other studies found null effects of provocativeness on victim blame, but the scenarios and measures used in these studies were quite different from those described above. Smith et al. (1976) manipulated provocativeness via the victim’s occupation (she was either a topless/bottomless dancer, social worker, or nun) and the scenario itself was prototypical of a stranger rape: the assault occurred while the victim was walking alone at night and a knife was used. Johnson (1995) also used an occupational manipulation of victim provocativeness, but asked only if the victim was *more* responsible than the perpetrator. This measure of victim blame is problematic given that participants generally indicate greater blame to the perpetrator than the victim (see Pollard, 1992; Grubb and Harrower, 2008; Landström et al., 2016).

The sexual history and experience of victims have also been considered as important contributors to victim blame (Whatley, 1996). This information is often manipulated via scenario descriptions of previous relationships or relationship status. Pugh (1983) manipulated the victim’s past sexual history via the victim’s testimony that she had or had not met other men in a bar and had sex with some of them prior to her alleged assault. When the victim was portrayed as more sexually promiscuous, she was blamed more for her assault (see also Kanekar and Seksaria, 1993; Idsis and Edoute, 2017). A “married mother of three” who went to a party and met a man who subsequently raped her (compared to a woman about whom no relationship information given), was found more to blame for her assault (Viki and Abrams, 2002). However, this manipulation may have less to do with sexual experience than with the perceived immorality of a married mother being at a party and flirting with a strange man.

Howells et al. (1984) found no differences in victim blame across their two levels of victim relationship status (single or engaged). However, in this scenario the victim was a family’s regular babysitter who was assaulted by her employer as he gave her a ride home. Participants’ schemas for babysitters as relatively young may have reduced overall victim blame in this case, as she was accosted by an older man in a position of power, which may have over-ridden any influence of relationship status on victim blaming tendencies.

Finally, only one study has manipulated the extent to which the victim’s sexual orientation influenced victim blaming (Ford et al., 1998). In this research a heterosexual female victim was found to be more at fault than a lesbian victim when assaulted by a male. This finding may speak to the “rape as sexual desire” myth mentioned previously, in that a heterosexual female may be seen as sexually enticing to the heterosexual male assailant (more likely to have “asked for it”) and therefore seen as more blameworthy than the lesbian victim.

#### Force and Resistance

The legal definition of rape includes mention of force, and thus, it is unsurprising that a majority of studies on acquaintance rape often include mention of force and/or victim resistance (80 of 102, see Table 1). For example, Shotland and Goodstein (1983) manipulated the amount of force the perpetrator used (verbal, or verbal and physical), the degree of victim resistance (verbal, or verbal and physical), and the onset timing of the victim’s resistance (immediately after a French kiss, after he begins to caress her below the waist, or after they are undressed). The type of resistance by the victim did not influence perceptions of victim blame (see also Sims et al., 2007), but perpetrator force in combination with onset of protest mattered. When low force was used, the victim was blamed regardless of when she began to protest. When the assailant was depicted as using both verbal and physical force, the victim was only blamed when she delayed protest until the point of undress. Similarly, Idsis and Edoute (2017) found victims were judged less responsible when they physically, rather than verbally, resisted, and when their resistance was depicted as strong, rather than weak. Other research indicates that victims are blamed less when the perpetrator uses physical force (e.g., see Bieneck and Krahé, 2011, although this study combined results across stranger, acquaintance, and ex-partner assaults). Victim resistance also appears to decrease victim blaming (Gilmartin-Zena, 1983; Black and Gold, 2008; Bongiorno et al., 2016, although these results are confounded as the more resistant victim was attacked by a stranger compared to a non-resisting acquaintance rape; dressed victim was attacked by a stranger; Kanekar and Seksaria, 1993; Masser et al., 2010, although this effect only occurred among those high in benevolent sexism that read about a victim who left her children unattended at home; McKimmie et al., 2014), especially when resistance occurs earlier in the encounter (Kopper, 1996). Perpetrator use of physical force also results in less victim blame than a case in which the victim is unable to resist due to intoxication (Krahé et al., 2007). While lacking an assessment of victim culpability, Branscombe and Weir (1992) found that *assailant* blame was highest when the victim strongly resisted physically (kicking him in the shin and fighting during the entire encounter compared to simply attempting to stand up). Degree of verbal resistance of the victim did not influence perceptions of perpetrator blame.

But some researchers have found different patterns. Wooten (1980) found greater victim blame when the perpetrator was depicted as using moderate force, compared to low or high force. However, this manipulation of force was confounded with victim resistance: In both the low force and high force conditions, victim resistance was both verbal and physical, but the moderate force condition depicted only verbal resistance. Still, this research suggests that victim resistance in combination with degree of force used by the assailant may be important for understanding blame (see also Shotland and Goodstein, 1983). The role of victim resistance may be particularly important among those who believe that rape is a sexually motivated crime (Ong and Ward, 1999). Compared to those who believed rape is motivated by power, participants who endorsed the belief that rape is sexually motivated blamed the victim more when she was described as not resisting the attack. When the victim did resist, however, these beliefs had no effect on victim blaming.

A meta-analysis on attribution of responsibility for accidents (not related to sexual assault) found that accident severity increased the tendency to blame the perpetrator (Burger, 1981). Therefore, an important component of force and resistance that should be assessed in future work is the severity of the assault in terms of both physical and emotional outcomes experienced by the victim. We located one study that manipulated whether the victim was physically hurt following the assault. There was no difference in blame, but participants did recommend longer prison sentences for the perpetrator when the victim was physically hurt (Kanekar et al., 1991; note: the impact of victim injury on sentencing was examined in two studies and was only significant in one). While the above findings on assault severity on blame appear to support the conclusions of the meta-analysis on blame for accidents, more research manipulating the severity of injury to the victim is needed.

#### Victim and Perpetrator Race

As noted in the discussion of race in the “individual factors” section, very little research has examined the role of victim and assailant race on blame in acquaintance rape cases; only five studies have investigated the role of victim/assailant race/ethnicity in victim blaming in acquaintance rape cases (see Table 1). This omission is problematic, given that more non-White women are victimized compared to White women (Black et al., 2011). Further, many myths surrounding sexual assault depict a Black male assailant and a White female victim (Davis, 1983; Epstein and Langenbahn, 1994). While lacking an assessment of victim blame, prior research manipulating both victim and perpetrator race in an acquaintance rape found that White victims were more likely than Black victims to prompt beliefs that the assailant should be held legally responsible and that his actions could be defined as criminal (Foley et al., 1995). Counter to the myth of the Black rapist, however, this research did not find any significant differences in blame based on assailant race. Willis (1992) found that regardless of race, victims were rated as less truthful in their reports of a sexual assault if they were depicted as being in a prior or current relationship with their Black assailant, compared to when he was depicted as White or as a Black stranger. Some evidence suggests greater victim blame in intra-racial compared to inter-racial rapes (George and Martinez, 2002), but this study collapsed across stranger and acquaintance rape scenarios (despite a main effect in which victims of acquaintance rape were blamed more than victims of stranger rape).

As discussed previously, cultural similarity to the perpetrator increased victim blaming among an Australian sample, but only if the victim was depicted as not resisting the attack (Bongiorno et al., 2016). Dupuis and Clay (2013) found that victim blame was a function of both the victim’s race and her perceived respectability, manipulated via the defendant’s testimony that the victim was either a “party girl” who often picked up men at bars or a “sweet girl” who didn’t date much or go to bars. While respectability did not matter for blame of White victims, it affected blame of Black victims: Respectable Black victims were blamed less than “party girl” Black victims. Furthermore, respectable Black victims were blamed less than respectable White victims, while “party girl” Black victims were blamed more than comparable White victims. Perpetrator race mattered only in one case– the non-respectable victim was seen as more blameworthy than the respectable victim when the perpetrator was Black.

These patterns may be complicated further by consideration of participant race. As described earlier, Varelas and Foley (1998) found that White participants blamed victims less than Black participants and that less blame was attributed to victims when the assailant was Black. White participants also blamed White victims assaulted by Black men less than Black victims assaulted by Black men, while Black participants attributed the most blame to a Black woman assaulted by a White man. One other study manipulated victim and assailant race (Willis, 1992) but did not report comparisons relevant comparisons.

Research on race effects has been limited by the singular focus on Black and White victims and perpetrators (but see Bell et al., 1994, for an exception). More research is needed on how other victim/assailant races (e.g., Asian, Hispanic) may influence blame, as well as potential interactions with participant demographics.

#### Socioeconomic Status

Sexual assault may be motivated by need for power (Brownmiller, 1975; Burt, 1980; Lonsway and Fitzgerald, 1994; Ward, 1995) and therefore power differentials within a rape scenario, defined by socioeconomic status, may influence evaluations of blame. Black and Gold (2008) manipulated socioeconomic status of the perpetrator by describing him as either a bus driver or doctor. Women, but not men, held the victim more responsible when she was assaulted by the bus driver than the doctor. In another study in which the victim was portrayed as either a cashier or accountant, both male and female participants rated the cashier as more promiscuous and more blameworthy (Spencer, 2016).

Blame may be more affected by the *relative status* of the perpetrator compared to the female victim. Using a sample of students at the University of Bombay, Kanekar et al. (1991) manipulated the assailant’s occupational status to be higher than, the same as, or lower than the status of the female victim, along with an additional manipulation of whether the victim filed a complaint or not against her aggressor. These researchers found a greater tendency for men to blame the victim when the assailant had higher status (or comparable status) relative to the victim, but only if she did not file a complaint. Relatedly, Yamawaki et al. (2007) manipulated whether the victim or assailant held a high status position or not (well-respected CEO versus student from a local university). When the assailant was in the more powerful position, those who believe women use sex to gain power from men blamed the victim more.

Drawing definitive conclusions from these studies about the effect of socioeconomic status on victim blame is difficult. Black and Gold (2008) did not provide information about the victim’s occupation and thus it is unclear whether participants assumed she held a better job than the bus driver, thus changing the power dynamic between the two. Yamawaki et al. (2007) did not include a control condition whereby the victim and assailant held equal power status. Finally, while Kanekar et al. (1991) found gender differences in blame due to relative status of the assailant, this only emerged in the conditions in which the victim chose not to file a complaint. Clearly more research is needed on socioeconomic status and other power differential cues to better determine their effects on victim blame.

### Summary of Situation Level Factors

Alcohol use is common in sexual assault cases and not surprisingly, a large number of sexual assault scenarios used in research include this feature. However, few studies have examined how changes in intoxication and alcohol use levels impact victim blame. Among those that have, the evidence largely suggests that alcohol use by the victim increases victim blaming, while alcohol use by the defendant reduces his level of blame (Richardson and Campbell, 1982; Stormo et al., 1997; Cameron and Stritzke, 2003; Bieneck and Krahé, 2011; but see Girard and Senn, 2008).

Research considering victim physical characteristics clearly indicates that the more revealing the clothing worn by the victim and the more suggestive her behavior or occupation, the more likely the victim is to be blamed for her assault (Muehlenhard and MacNaughton, 1988; Kanekar and Seksaria, 1993; Cassidy and Hurrell, 1995; Workman and Orr, 1996; Loughnan et al., 2013). Victims with an apparently promiscuous sexual history are also found to be more blameworthy (Pugh, 1983). Provocativeness may also interact with participant gender, such that men, but not women blame provocatively dressed victims more than conservatively dressed victims (Whatley and Riggio, 1992). In one study on victim sexual orientation, heterosexual victims were blamed more than lesbian victims (Ford et al., 1998). Many of these findings are consistent with the belief that physical enticement—based on dress, history, or sexual orientation — triggers assault, but one exception to this pattern is the finding that unattractive victims are blamed more than attractive victims (Gerdes et al., 1988). The latter finding may have more to do with a general halo effect favoring attractive individuals (e.g., Dion et al., 1972).

Another common factor considered in sexual assault vignettes is the degree of force and resistance used by the perpetrator and victim. These appear to play an important role in perceptions of victim culpability. Victims who resist their attackers are seen as less blameworthy than those who do not (particularly when they resist early in the interaction; Shotland and Goodstein, 1983; Kanekar and Seksaria, 1993; Kopper, 1996; Black and Gold, 2008). Less victim blaming also occurs when the perpetrator is depicted as using a great degree of force (Bieneck and Krahé, 2011) and when the victim is portrayed as having been injured from the attack (Kanekar et al., 1991).

Despite evidence that non-White women are more likely to be victimized (Black et al., 2011), there is currently relatively little research that manipulates victim and perpetrator race. The work that has been done, however, indicates a more complex interaction with other individual and situational factors. For instance, White participants blamed White victims assaulted by Black men less than Black victims assaulted by Black men, while Black participants attributed the most blame to a Black woman assaulted by a White man (Varelas and Foley, 1998). Further, respectability mattered for blame of Black victims, but not White victims.

Finally, research on the impact of socioeconomic status and power differences between victim and assailant is currently too limited and inconsistent to draw definitive conclusions. However, some research points to the importance of power differentials in influencing blame (Kanekar et al., 1991), and of participants’ beliefs that women use sex to gain power from men (Yamawaki et al., 2007).

One difficulty in assessing the impact of situational factors on victim blame is that many published studies do not include full descriptions of the scenarios used. For instance, the sexual assault scenario used by Janoff-Bulman et al. (1985) is simply described as a “first person account of a rape and the events preceding it (pp. 164).” After having received the full scenario by Dr. Janoff-Bulman, however, it is clear that alcohol intoxication played a central role in this scenario (“I had more than I could handle. Bob got drunk too…I had a lot to drink…I insisted we stay until we had…something to get more sober”). Given the role alcohol plays in evaluations of sexual assault, it is important to be aware that this sexual assault scenario centers around a night of heavy drinking. Thus, before we can draw firm conclusions about the effects of various situational factors on victim blame, access to the full scenarios used in research is necessary.

## Discussion

We have reviewed a variety of individual and situational factors that influence victim blaming, but in order to fully understand victim blame we must take into account broader institutional and societal factors that may dictate how perceivers view any given sexual assault scenario. Indeed, it has been suggested that the only way to truly prevent rape is to address the problem of rape at the societal level (Allison and Wrightsman, 1993), considering broader cultural factors that both contribute to sexual assaults and promote rape myths and victim blaming.

As depicted in Figure 1, we view individual, situational, and institutional factors as influencing one another. Interactions within individual level factors (e.g., participant gender and rape myth endorsement), situational level factors (e.g., perpetrator force and assailant resistance) and the interaction between individual and situation level factors (e.g., participant race and victim/assailant race) have received some consideration in the research literature. What has yet to be accounted for, however, is how these elements may also be influenced by the cultural context in which they are studied. In the following sections we identify institutional-level factors that may contribute to both sexual assault and victim blaming and then discuss how these factors may interact with individual and situation level factors.

### Institutional/Societal Level Factors

#### Gender Dynamics

Patriarchy is widespread across many cultures (Pratto, 1996; Eagly and Wood, 1999; World Economic Forum, 2017) and feminist scholars have long proposed that sexual assault is motivated by power, with violence against women a function of gendered sex roles that support male domination and female exploitation (Brownmiller, 1975; Burt, 1980; Lonsway and Fitzgerald, 1994; Ward, 1995). Societies that have more egalitarian gender roles tend to have lower rates of sexual assault (Sanday, 1981; White et al., 1997). Interestingly, recent work has found that priming men to feel lower in power increases their ability to take others’ perspective, thereby decreasing their tendency to blame victims of acquaintance rape (Gravelin et al., 2017).

Socialization into gender roles may make women more prone to the dangers of sexual assault, but also communicates victim blaming as normative. For instance, Warshaw (1994) argues that communal roles teach women from a young age to avoid embarrassing a man by rejecting his advances and to not resist a physically aggressive man. Male gender roles may also justify and promote sexually aggressive behavior among men (Griffin, 1971; Sanday, 1981; Beneke, 1982; Warshaw, 1994; O’Toole, 2007) and legitimize victim blaming (Griffin, 1971; Check and Malamuth, 1983; Margolin et al., 1989; Feltey et al., 1991). For example, men may be taught to dissociate themselves from responsibility for their sexual actions, thereby reinforcing myths that once a man is sexually aroused he cannot stop himself (Warshaw, 1994).

Stereotypes and sexual scripts communicated to men and women further complicate sexual relations. Considerable research documents a sexual double standard, whereby men are more free than women to express their sexual desires (Sprecher et al., 1987; Muehlenhard and Hollabaugh, 1988; Muehlenhard and McCoy, 1991; Muehlenhard and Quackenbush, 1998). This pattern reinforces a common belief in token resistance, whereby it is thought that many women say no to sex even when they would like to say yes since it is “unladylike” to desire sex (Gagnon and Simon, 1973; Check and Malamuth, 1983; Schur, 1983; Muehlenhard and Hollabaugh, 1988; Warshaw, 1994). This belief appears to influence approaches to sexual behavior; Muehlenhard and Hollabaugh (1988) found that over 39% of their sample of undergraduate women reported engaging in token resistance at least once, and those who had were more likely to endorse traditional gender roles than women who were sexually active but did not engage in token resistance.

Men are socialized to be the sexual initiators, and, given the belief in, and practice of, token resistance, may be encouraged not to take a woman’s reluctance seriously. Thus, sex is often viewed as a challenge, and women become sexualized objects to conquer (Warshaw, 1994). These sexual scripts dictating token resistance from women and persistence by men ambiguate what is viewed as sexual foreplay and what is sexual assault. Acceptance of such scripts may also influence perceivers’ evaluation of acquaintance rape victims who resist sexual advances from the assailant. Indeed, research has established that endorsement of gender inequality and traditional gender roles (which includes the practice of token resistance, Muehlenhard and Hollabaugh, 1988) is associated with greater RME and victim blaming (Brownmiller, 1975; Burt, 1980; Deitz et al., 1982; Whatley, 2005; Edwards et al., 2011).

Another strong cultural force that dictates what is considered proper gender role and sexual behavior is religion. A variety of religions, such as Christian evangelism and Islam promote a gender hierarchy that values female submission (see Flood and Pease, 2009); other religious affiliations may convey more or less conservatism regarding appropriate sexual behavior. Using 20 years of data from the General Social Survey, Hoffman and Miller (1997) found that more conservative religions promote traditional female roles while liberal religions promote egalitarianism. Further, the strength of gender norms in a given culture may interact with individual level factors to influence evaluations of victim blame. For example, the extent to which sexually promiscuous victims are blamed may be exacerbated in conservative religious cultures. More generally, to the extent that institutions promote a gendered hierarchy, men possessing lower social power than women are likely to feel threatened, which in turn may lead to more victim blame (e.g., Munsch and Willer, 2012).

#### Media and Sexual Objectification

The hypersexualization and sexual objectification of women in society also leads to greater acceptance of violence against women and victim blame (Malamuth and Check, 1981; Ohbuchi et al., 1994; Lanis and Covell, 1995; MacKay and Covell, 1997; Kalof, 1999). Hypersexualization and sexual objectification refer to the extreme sexuality ascribed to women, often depicting them as purely sexual objects for men’s desires. This sexualized representation of women exists in a variety of domains, including pornography, non-pornographic film and television, and print advertising (see Stankiewicz and Rosselli, 2008).

Not only do media outlets often depict women as sexualized objects, but sexual aggression is portrayed as normative behavior in pornography (Longino, 1980; MacKinnon, 1985), films (Donnerstein and Linz, 1986), and music (Schur, 1988; hooks, 1994). While victims of non-sexual aggression are often shown as having suffered from their assault, sexual assault victims are often depicted as initially refusing a man’s sexual advances and then become aroused as he ignores her resistance (Smith, 1976; Zilbergeld, 1978; Malamuth and Check, 1981). Eroticizing sexual dominance in the media legitimizes violence against women and may contribute to victim blaming (see Schur, 1988).

In the context of sexual assault, the media also tend to focus on stranger rape (Soothill, 1991), thus influencing how perceivers determine what constitutes a “real rape,” and to portray rapists as strangers with solely sexual motivations to assault attractive young females (Allison and Wrightsman, 1993). Deviations from this image to one depicting an acquaintance rape may be less likely to be seen as a sexual assault, resulting in increased victim blaming. Soothill (1991) documented changes in reporting on sexual assaults in major newspapers from 1973 to 1985. Despite an increase in the number of single assailant-single victim sexual assault crimes in the courts across this period, reporting on these types of crimes decreased, with a shift in focus to multiple offender gang rapes instead. This shift may have increased readers’ beliefs that gang rapes and stranger rapes are more prevalent and concerning than acquaintance rape. A more recent review of two major newspapers’ reporting on sexual assault indicates that gang and stranger rapes are still over-reported relative to acquaintance rapes and to actual prevalence data (Gravelin, 2017).

When media outlets do discuss acquaintance rape, *how* it is discussed can also contribute to victim blaming. Highlighting rape myths or focusing on ways that acquaintance rapes may resemble prototypical stranger rapes may have negative consequences for victims of assaults that do not include these prototypical features. For example, Franiuk et al. (2008) exposed participants to headlines about an acquaintance rape case against basketball star Kobe Bryant. These headlines were modeled after actual headlines used in newspaper accounts of Bryant’s case and either contained rape myths (e.g., “Defense attorneys in sexual assault case say accuser had motive to lie”) or not (“Hearing set for man accused of sexual assault”). Participants tended to see Bryant as less guilty after reading headlines containing rape myths than neutral headlines, and this was particularly true among men. Men exposed to the rape myth headlines also endorsed rape-supportive attitudes more so than men in the control condition. In short, the media may exacerbate endorsement of rape myths, which in turn promotes greater victim blaming.

#### Legal and Empirical Rhetoric

The definition of rape has changed throughout American history and therefore what constitutes rape is dependent on the time and state in which the assault has occurred (Freedman, 2013). It was not until 2012 that the FBI broadened the definition of rape to include *non-forcible* rape of women *and men*. In 2014, both California and New York altered their definitions of sexual assault such that rape is not defined by the victim saying “no,” but by failing to say “yes.” Such a definition acknowledges the role of the assailant in obtaining affirmative consent, rather than the victim in saying no. Branscombe et al. (1996); see also Nario-Redmond and Branscombe (1996) found that focusing participants on how the victim’s behavior could have altered a rape outcome produced the greatest amount of victim blame, while focusing on how the assailant’s behavior could have prevented an assault generally increased the relative blame assigned to him. Others have found that defining sexual assault as an act of intergroup (a “hate crime”), rather than interpersonal violence (a personal assault) reduced victim blaming in both stranger and acquaintance rape cases (Droogendyk and Wright, 2014).

Despite these recent efforts to broaden the definition of rape and incorporate definitions more closely aligned with non-stranger rape, earlier constructions of rape promoted through rape myths remain deeply embedded in our culture. These myths make it difficult for individuals to recognize rape, particularly non-stranger rape. This difficulty may encourage perceivers to look to situational factors such as the victim’s attractiveness and promiscuity to explain the assault in acquaintance rape cases (Weis and Borges, 1973). Given that the working definition of what constitutes a rape varies as a function of time and location, comparing studies conducted in different settings at different times may not be appropriate.

#### Rape Culture

Much research on acquaintance rape asserts that certain settings foster beliefs conducive to rape, often referred to as “rape culture” (Buchwald et al., 1993). Some have suggested that individuals within the United States as a whole view rape as normative and a condoned behavior (Rozée, 1993; Koss et al., 1994), but rape culture is most often associated with college campuses, particularly athletic groups and fraternities. Rape cultures exist outside of the college environment, as well; both high school and professional-level athletics and the military have been studied as rape cultures (see O’Toole, 2007).

Researchers suggest that male-dominated environments such as those mentioned above are particularly likely to promote sexist attitudes and behaviors and may facilitate greater risk of sexual assault as well as victim-blaming myths (Sanday, 1990; Melnick, 1992; Koss and Gaines, 1993; Boeringer, 1996, 1999; Boswell and Spade, 1996; Bleecker and Murnen, 2005; McCray, 2014). Rape cultures are typically defined as hypermasculinized environments that glorify coercive sexual behavior as central to their group identity (O’Toole, 2007). For example, all-male housing units such as fraternities have a higher risk of sexual assaults than co-ed housing (Hinch and Thomas, 1999). Sexual aggression is also particularly likely among the newest members of an all-male group: Fraternity pledges are the most likely of all college males to commit a sexual assault on campus (see Bohmer and Parrot, 1993). Individual level factors such as threats to power or status may be particularly problematic within all-male groups, increasing the likelihood of sexual assault, rape myth endorsement, and victim blaming.

Rape culture is maintained by the norm of silencing victims of rape (Burnett et al., 2009). Particularly in cultures where rape myths are promoted and accepted, victims may question their behavior and be uncertain whether to label their experience as a rape or not (Adams-Curtis and Forbes, 2004; Harned, 2004). Failure to report rape not only protects perpetrators from punishment but also communicates a tolerance for sexual assault that delegitimizes victims’ experiences and perpetuates victim blaming.

Rape culture frameworks tend to focus on localized settings that contribute to sexual assault and victim blaming, but broader cultural contexts—including national and regional contexts—have differing historical experience with violence and differing flexibility or rigidity of gender roles which may contribute to differing levels of victim blame (Sanchez-Hucles and Dutton, 1999). A qualitative study on community norms and expectations concerning intimate violence by Sorenson (1996) found that compared to Asian American participants, Mexican American participants described a greater cultural value on male sexual prowess. Victims of sexual assault in many Middle Eastern communities are punished, even outcast by their families, or must marry their rapists in order to restore honor to their families (Ruggi, 1998). Conversely, many African cultures promote flexible gender roles and pride in having strong, independent women, thus potentially reducing blame ascribed to female victims who deviate from traditional gender roles (Hill, 1972; Young, 1986; Boyd-Franklin, 1989, see also Sanchez-Hucles and Dutton, 1999). Finally, Ho (1990), see also Sorenson (1996) noted that Asian values of harmony and close family ties may not promote lesser sexual violence, but may support minimizing or concealing violence.

These cultural differences may contribute both to differences in sexual assault rates and differing levels of victim blaming. A report by the World Health Organization [WHO] (2005) compiled cross-national data from surveys on female victimization from 1992 through 1997 and found considerable variability in reported victimization. For instance, Asian countries (China, India, Indonesia, and Philippines) had the lowest rate of reported sexual assault as well as the lowest variability within-continent, with incidence of sexual assault ranging from 0.3% in the Philippines, to 2.7% in Indonesia, while the data surveyed from countries in Latin America (Argentina, Bolivia, Brazil, Columbia, Costa Rica, and Paraguay) had the largest variability, with incidences ranging from ranging from 1.4% in Bolivia to 8.0% in Brazil. It is important to note that, while informative, these data do not distinguish between types of sexual assault, and sample sizes varied considerably across studies. Respondents were only asked about sexual assaults that had occurred within the last 5 years and thus does not account incidents outside of this window. There is no national data base on victim blaming, but differing cultural tendencies to minimize or silence sexual assault may communicate greater victim blame by way of trivializing experiences of sexual assault.

Another element varying across regional/ethnic cultures that may contribute to differential evaluations of victims of sexual assault is religiosity; cultures vary in the extent to which they are influenced by religious doctrines. While limited to a sample of undergraduates living in the United States, a study on the role of cultural and religious influences on endorsement of traditional gender roles found more conservative sexual attitudes among Asians (South and East Asians) compared to their Hispanic (South American, Central American, and Mexican) and European American (Caucasian) counterparts (Ahrold and Meston, 2010). Across all three groups, greater intrinsic religiosity and religious fundamentalism predicted more conservative sexual attitudes (endorsement of traditional gender roles). Thus, religiosity and traditional gender role endorsement attitudes may interact with situational elements to contribute to differential degrees of victim blaming. For example, a victim who deviates from a traditional submissive role by behaving promiscuously or fighting her attacker may be seen as more blameworthy by more religious and conservative observers.

## Final Remarks

Research on sexual assault and victim blaming is burgeoning, yet much more needs to be done to understand the individual, situational, and cultural factors that contribute to victim blaming, particularly in the case of acquaintance rape. The current paper identified the most commonly studied aspects of victim blaming in acquaintance rape within the two primary approaches: individual level factors and situation level factors. A review of this literature reveals many inconsistent findings and interactions across both levels. In an effort to make sense of these complex interactions and inconsistent findings, we suggest greater consideration be given to the role of institutional factors on evaluations of victim blame. The final sections of this paper then outlined various institutional factors that we believe should be given greater attention in future research on victim blaming in acquaintance rape and provided evidence to support why these factors may interact with the more commonly studied individual and situational factors.

Acquaintance rapes differ in many ways and therefore researchers cannot use a “standardized” single vignette to study victim blame. However, knowing which details are present or absent in the scenarios used by researchers will help in drawing more accurate and appropriate comparisons and conclusions. Further, despite obvious differences between acquaintance and stranger rape, many researchers still use findings gathered from one type of assault interchangeably with the other when discussing patterns in sexual assault research (Whatley, 1996; Grubb and Harrower, 2008; Grubb and Turner, 2012). As previously highlighted, a substantial number of the papers considered in this review failed to provide full details of the scenarios used in their research. As elements such as the presence/absence of alcohol, victim’s clothing and promiscuity, and prior relationship with the assailant all influence how perceivers evaluate cases of sexual assault, it is important to be aware of the full characterization of the sexual assault before drawing conclusions across studies. Therefore, in addition to accounting for institutional factors in future examinations of victim blaming, greater transparency about and open sharing of the scenarios used is needed.

Our narrative review allowed for a wide-ranging overview of research on victim blame in acquaintance rape cases but was limited by a reliance on study significance levels, without taking into account study power (i.e., low *N* and high *N* studies received equal weight in our review). This limitation can be redressed by the use of meta-analysis to better quantify the effects of individual, situational, and cultural factors on victim blaming. We hope this review motivates such meta-analytic consideration, as well as additional original research in these areas. The #MeToo movement has brought recent heightened public attention to the problem of sexual assault; this cultural focus may further spur social scientific efforts toward understanding perceptions and treatment of victims of sexual assault.

## Author Contributions

CG served as primary investigator, responsible for conception of analysis and review, literature collection, synthesis, critique and write up. MB provided substantial contributions to synthesis and critique and assisted in multiple revisions of manuscript document. CB assisted in literature collection and synthesis/coding and aided in final revisions of manuscript.

## Conflict of Interest Statement

The authors declare that the research was conducted in the absence of any commercial or financial relationships that could be construed as a potential conflict of interest.

## References

[B1] Abbey A. Ross L. T. McDuffie D. McAuslan P. (1996). Alcohol and dating risk factors for sexual assault among college women. *Psychol. Women Q.* 20 147–169. 10.1111/j.1471-6402.1996.tb00669.x

[B2] Abrams D. Viki G. T. Masser B. Bohner G. (2003). Perceptions of stranger and acquaintance rape: the role of benevolent and hostile sexism in victim blame and rape proclivity. *J. Pers. Soc. Psychol.* 84 111–125. 10.1037/0022-3514.84.1.111 12518974

[B3] Adams-Curtis L. E. Forbes G. B. (2004). College women’s experiences of sexual coercion: a review of cultural, perpetrator, victim, and situational variables. *Trauma Violence Abuse* 5 91–122. 10.1177/1524838003262331 15070552

[B4] Ahrold T. K. Meston C. M. (2010). Ethnic differences in sexual attitudes of U.S. college students: gender, acculturation, and religiosity factors. *Arch. Sex. Behav.* 39 190–202. 10.1007/s10508-008-9406-1 18839302 PMC4426857

[B5] Allison J. A. Wrightsman L. S. (1993). *Rape: The Misunderstood Crime.* Thousand Oaks, CA: Sage Publications.

[B6] Amir M. (1971). *Patterns in Forcible Rape.* Chicago, IL: University of Chicago Press.

[B7] Anderson K. B. Cooper H. Okamura L. (1997). Individual differences and attitudes toward rape: a meta-analytic review. *Pers. Soc. Psychol. Bull.* 23 295–315. 10.1177/0146167297233008

[B8] Ayala E. E. Kotary B. Hetz M. (2015). Blame attributions of victims and perpetrators: effects of victim gender, perpetrator gender, and relationship. *J. Interpers. Violence* 33 94–116. 10.1177/0886260515599160 26264724

[B9] Basow S. A. Minieri A. (2011). “You owe me”: effects of date cost, who pays, participant gender, and rape myth beliefs on perceptions of rape. *J. Interpers. Violence* 26 479–497. 10.1177/0886260510363421 20442451

[B10] Bell S. T. Kuriloff P. J. Lottes I. (1994). Understanding attributions of blame in stranger rape and date rape situations: an examination of gender, race, identification, and students’ social perceptions of rape victims. *J. Appl. Soc. Psychol.* 24 1719–1734. 10.1111/j.1559-1816.1994.tb01571.x

[B11] Ben-David S. Schneider O. (2005). Rape perceptions, gender role attitudes, and victim-perpetrator acquaintance. *Sex Roles* 53 385–399. 10.1007/s11199-005-6761-4 29518275

[B12] Beneke T. (1982). *Men on Rape.* New York, NY: St. Martin’s Press.

[B13] Benson B. J. Gohm C. L. Gross A. M. (2007). College women and sexual assault: the role of sex-related alcohol expectancies. *J. Fam. Violence* 22 341–351. 10.1007/s10896-007-9085-z 28933955

[B14] Bieneck S. Krahé B. (2011). Blaming the victim and exonerating the perpetrator in cases of rape and robbery: is there a double standard? *J. Interpers. Violence* 26 1785–1797. 10.1177/0886260510372945 20587449

[B15] Black K. A. Gold D. J. (2008). Gender differences and socioeconomic status biases in judgments about blame in date rape scenarios. *Violence Vict.* 23 115–128. 10.1891/0886-6708.23.1.115 18396585

[B16] Black M. C. Basile K. C. Breiding M. J. Smith S. G. Walters M. L. Merrick M. T. (2011). *The National Intimate Partner and Sexual Violence Survey (NISVS): 2010 Summary Report.* Atlanta, GA: Centers for Disease Control and Prevention.

[B17] Bleecker E. T. Murnen S. K. (2005). Fraternity membership, the display of degrading sexual images of women, and rape myth acceptance. *Sex Roles* 53 487–493. 10.1007/s11199-005-7136-6

[B18] Blumberg M. L. Lester D. (1991). High school and college students’ attitudes toward rape. *Adolescence* 26 727–729. 1962554

[B19] Boeringer S. B. (1996). Influences of fraternity membership, athletics, and male living arrangements on sexual aggression. *Violence Against Women* 2 134–147. 10.1177/1077801296002002002 12295455

[B20] Boeringer S. B. (1999). Associations of rape-supportive attitudes with fraternal and athletic participation. *Violence Against Women* 5 81–90. 10.1177/10778019922181167 31454873

[B21] Bohmer C. Parrot A. (1993). *Sexual Assault on Campus: The Problem and the Solution.* New York, NY: Lexington Books.

[B22] Bongiorno R. McKimmie B. M. Masser B. M. (2016). The selective use of rape-victim stereotypes to protect culturally similar perpetrators. *Psychol. Women Q.* 40 398–413. 10.1177/0361684316631932

[B23] Boswell A. A. Spade J. Z. (1996). Fraternities and collegiate rape culture: why are some fraternities more dangerous places for women? *Gend. Soc.* 10 133–147. 10.1177/089124396010002003

[B24] Boyd-Franklin N. (1989). *Black Families in Therapy: A Multisystems Approach.* New York, NY: Guilford Press.

[B25] Bradbury T. N. Fincham F. D. (1990). Attributions in marriage: review and critique. *Psychol. Bull.* 107 3–33. 10.1037/0033-2909.107.1.3 2404292

[B26] Branscombe N. R. Owen S. Garstka T. A. Coleman J. (1996). Rape and accident counterfactuals: who might have done otherwise and would it have changed the outcome? *J. Appl. Soc. Psychol.* 26 1042–1067. 10.1111/j.1559-1816.1996.tb01124.x

[B27] Branscombe N. R. Weir J. A. (1992). Resistance as stereotype-inconsistency: consequences for judgments of rape victims. *J. Soc. Clin. Psychol.* 11 80–102. 10.1521/jscp.1992.11.1.80 18396585

[B28] Bridges J. S. (1991). Perceptions of date and stranger rape: a difference in sex-role expectations and rape-supportive beliefs. *Sex Roles* 24 291–307. 10.1007/BF00288303

[B29] Bridges J. S. McGrail C. A. (1989). Attributions of responsibility for date and stranger rape. *Sex Roles* 21 273–286. 10.1007/BF00289907 15005999

[B30] Brownmiller S. (1975). *Against Our Will: Men, Women and Rape.* New York, NY: Simon & Schuster.

[B31] Buchwald E. Fletcher P. Roth M. (1993). “Are we really living in a rape culture,” in *Transforming a Rape Culture*, eds Buchwald E. Fletcher P. Roth M. (Minneapolis, MN: Milkweed Editions), 7–10.

[B32] Burger J. M. (1981). Motivational biases in the attribution of responsibility for an accident: a meta-analysis of the defensive-attribution hypothesis. *Psychol. Bull.* 90 496–512. 10.1037/0033-2909.90.3.496

[B33] Burnett A. Mattern J. L. Herakova L. L. Kahl D. H. Tobola C. Bornsen S. E. (2009). Communicating/muting date rape: a co-cultural theoretical analysis of communication factors related to rape culture on a college campus. *J. Appl. Commun. Res.* 37 465–485. 10.1080/00909880903233150

[B34] Burt M. B. (1980). Cultural myths and supports for rape. *J. Pers. Soc. Psychol.* 38 217–230. 10.1037/0022-3514.38.2.217 7373511

[B35] Calhoun K. S. Townsley R. M. (1991). “Attributions of responsibility for acquaintance rape,” in *Acquaintance Rape: The Hidden Crime*, eds Parrot A. Bechhofer L. (New York, NY: John Wiley), 57–69.

[B36] Calhoun L. G. Selby J. W. Warring L. J. (1976). Social perception of the victim’s causal role in rape: an exploratory examination of four factors. *Hum. Relat.* 29 517–526. 10.1177/001872677602900602

[B37] Cameron C. A. Stritzke W. G. K. (2003). Alcohol and acquaintance rape in Australia: testing the presupposition model of attributions about responsibility and blame. *J. Appl. Soc. Psychol.* 33 983–1008. 10.1111/j.1559-1816.2003.tb01935.x

[B38] Casarella-Espinoza M. (2015). *Whose Fault is it Anyway? Comparison of Victim Blaming Attitudes Towards Sex Trafficking and Sexual Assault Across Gender and Two Ethnic Groups (Order No. AAI3639712).* Available at: http://search.proquest.com/docview/1705044617?accountid=14556

[B39] Cassidy L. Hurrell R. M. (1995). The influence of victim’s attire on adolescents’ judgments of date rape. *Adolescence* 30 319–323. 7676869

[B40] Check J. V. Malamuth N. M. (1983). Sex role stereotyping and reactions to depicting stranger versus acquaintance rape. *J. Pers. Soc. Psychol.* 45 344–356. 10.1037/0022-3514.45.2.344

[B41] Coller S. A. Resick P. A. (1987). Women’s attributions of responsibility for date rape” The influence of empathy and sex-role stereotyping. *Violence Vict.* 2 115–125. 10.1891/0886-6708.2.2.115 3154159

[B42] Cowan G. Quinton W. (1997). Cognitive style and attitudinal correlates of the perceived causes of rape scale. *Psychol. Women Q.* 21 227–245. 10.1111/j.1471-6402.1997.tb00110.x

[B43] Critchlow B. (1985). The blame in the bottle: attributions about drunken behavior. *Pers. Soc. Psychol. Bull.* 11 258–274. 10.1177/0146167285113003

[B44] Davis A. Y. (1983). *Rape, Racism, and the Myth of the Black Rapist. Women, Race, and Class.* New York, NY: Vintage Books, 151–175.

[B45] D’Cruz J. Kanekar S. (1992). Attribution of fault to a rape victim as a function of the attributor’s celibate or married lifestyle. *Ir. J. Psychol.* 13 283–294. 10.1080/03033910.1992.10557888

[B46] Deitz S. R. Blackwell K. T. Daley P. C. Bentley B. J. (1982). Measurement of empathy toward rape victims and rapists. *J. Pers. Soc. Psychol.* 43 372–384. 10.1037/0022-3514.43.2.372 7120042

[B47] Dion K. Berscheid E. Walster E. (1972). What is beautiful is good. *J. Pers. Soc. Psychol.* 24 285–290. 10.1037/h0033731 4655540

[B48] Donnerstein E. Berkowitz L. (1981). Victim reactions in aggressive erotic films as a factor in violence against women. *J. Pers. Soc. Psychol.* 41 710–724. 10.1037/0022-3514.41.4.710 7288566

[B49] Donnerstein E. Linz D. (1986). Mass media sexual violence and male viewers current theory and research. *Am. Behav. Sci.* 29 601–618. 10.1177/000276486029005007

[B50] Droogendyk L. Wright S. C. (2014). Perceptions of interpersonal versus intergroup violence: the case of sexual assault. *PLoS One* 9:e112365. 10.1371/journal.pone.0112365 25419567 PMC4242515

[B51] Dull R. T. Giacopassi D. J. (1987). Demographic correlates of sexual and dating attitudes: a study of date rape. *Crim. Justice Behav.* 14 191–212. 10.1177/0093854887014002004

[B52] Dupuis E. C. Clay J. A. (2013). The role of race and respectability in attributions of responsibility for acquaintance rape. *Violence Vict.* 28 1085–1095. 10.1891/0886-6708.VV-D-12-00013 24547682

[B53] Eagly A. H. Diekman A. B. Johannesen-Schmidt M. C. Koenig A. M. (2004). Gender gaps in sociopolitical attitudes: a social psychological analysis. *J. Pers. Soc. Psychol.* 87 796–816. 10.1037/0022-3514.87.6.796 15598107

[B54] Eagly A. H. Wood W. (1999). The origins of sex differences in human behavior: evolved dispositions versus social roles. *Am. Psychol.* 54 408–423. 10.1037/0003-066X.54.6.408

[B55] Edwards K. M. Turchik J. A. Dardis C. M. Reynolds N. Gidycz C. A. (2011). Rape myths: history, individual and institutional-level presence, and implications for change. *Sex Roles* 65 761–773. 10.1007/s11199-011-9943-2

[B56] Eigenberg H. Garland R. (2008). “Victim blaming,” in *Controversies in Victimology*, ed. Moriarty L. J. (Newark, NJ: Elsevier Press), 21–36.

[B57] Epstein J. Langenbahn S. (1994). *Criminal Justice & Community Response to Rape.* Washington, DC: DIANE Publishing.

[B58] Estrich S. (1987). *Real Rape.* Cambridge, MA: Harvard University Press.

[B59] Ewoldt C. A. Monson C. M. Langhinrichsen-Rohling J. (2000). Attributions about rape in a continuum of dissolving marital relationships. *J. Interpers. Violence* 15 1175–1182. 10.1177/088626000015011004

[B60] Feild H. S. (1978). Attitudes toward rape: a comparative analysis of police, rapists, crisis counselors, and citizens. *J. Pers. Soc. Psychol.* 36 156–179. 10.1037/0022-3514.36.2.156

[B61] Feltey K. M. Ainslie J. J. Geib A. (1991). Sexual coercion attitudes among high school students: the influence of gender and rape education. *Youth Soc.* 23 229–250. 10.1177/0044118X91023002004

[B62] Ferrão M. C. Gonçalves G. Giger J. Parreira T. (2016). Judge me, judge me not: the role of eye size and observer gender on acquaintance rape. *An. Psicol.* 32 241–249. 10.6018/analesps.32.1.185701

[B63] Ferro C. Cermele J. Saltzman A. (2008). Current perceptions of marital rape: some good and not-so-good news. *J. Interpers. Violence* 23 764–779. 10.1177/0886260507313947 18272725

[B64] Fischer G. J. (1987). Hispanic and majority student attitudes toward forcible date rape as a function of differences in attitudes toward women. *Sex Roles* 17 93–101. 10.1007/BF00287902

[B65] Fisher B. S. Cullen F. T. Turner M. G. (2000). *The Sexual Victimization of College Women.* Washington, DC: National Institute of Justice, 10.1037/e377652004-001

[B66] Fisher B. S. Daigle L. E. Cullen F. T. Turner M. G. (2003). Acknowledging sexual victimization as rape: results from a national–level study. *Justice Q.* 20 535–570. 10.1080/07418820300095611

[B67] Flood M. Pease B. (2009). Factors influencing attitudes to violence against women. *Trauma Violence Abuse* 10 125–142. 10.1177/152483800933413 19383630

[B68] Foley L. A. Evancic C. Karnik K. King J. Parks A. (1995). Date rape: effects of race of assailant and victim and gender of subjects on perceptions. *J. Black Psychol.* 21 6–18. 10.1177/00957984950211002

[B69] Ford T. M. Liwag-McLamb M. G. Foley L. A. (1998). Perceptions of rape based on sex and sexual orientation of victim. *J. Soc. Behav. Pers.* 13 253–263.

[B70] Franiuk R. Seefelt J. L. Vandello J. A. (2008). Prevalence of rape myths in headlines and their effects on attitudes toward rape. *Sex Roles* 58 790–801. 10.1007/s11199-007-9372-4

[B71] Freedman E. B. (2013). *Redefining Rape: Sexual Violence in the Era of Suffrage and Segregation.* Cambridge, MA: Harvard University Press.

[B72] Frese B. Moya M. Megias J. L. (2004). Social perception of rape: how rape myth acceptance modulates the influence of situational factors. *J. Interpers. Violence* 19 143–161. 10.1177/0886260503260245 15005999

[B73] Fulero S. M. DeLara C. (1976). Rape victims and attributed responsibility: a defensive attribution approach. *Victimology* 1 551–563.

[B74] Gagnon J. H. Simon W. (1973). “Youth, sex, and the future,” in *Youth in Contemporary Society*, ed. Gottlieb D. (Oxford: Sage).

[B75] Geiger B. Fischer M. Eshet Y. (2004). Date-rape supporting and victim-blaming attitudes among high school students in a multiethnic society: Israel. *J. Interpers. Violence* 19 406–426. 10.1177/0886260503262080 15038882

[B76] George W. H. Martinez L. J. (2002). Victim blaming in rape: effects of victim and perpetrator race, type of rape, and participant racism. *Psychol. Women Q.* 26 110–119. 10.1111/1471-6402.00049

[B77] Gerdes E. Dammann E. Heilig K. (1988). Perceptions of rape victims and assailants: effects of physical attractiveness, acquaintance, and subject gender. *Sex Roles* 19 141–153. 10.1007/BF00290151

[B78] Giacopassi D. J. Dull R. T. (1986). Gender and racial differences in the acceptance of rape myths within a college population. *Sex Roles* 15 63–75. 10.1007/BF00287532

[B79] Gilmartin-Zena P. (1983). Attribution theory and rape victim responsibility. *Deviant Behav.* 4 357–374. 10.1080/01639625.1983.9967622 24052599

[B80] Girard A. L. Senn C. Y. (2008). The role of the new “date rape drugs” in attributions about date rape. *J. Interpers. Violence* 23 3–20. 10.1177/0886260507307648 18087029

[B81] Glick P. Fiske S. T. (2001). An ambivalent alliance: hostile and benevolent sexism as complementary justifications for gender inequality. *Am. Psychol.* 56 109–118. 10.1037//0003-066X.56.2.109 11279804

[B82] Gordon M. T. Riger S. (2011). *The Female Fear.* New York, NY: The Free Press.

[B83] Gravelin C. R. (2017). *Assessing the Impact of Media on Blaming the Victim of Acquaintance Rape.* Doctoral Dissertation, University of Kansas, Lawrence, KS.

[B84] Gravelin C. R. Baldwin M. W. Biernat M. (2017). The impact of power and powerlessness on blaming the victim of sexual assault. *Group Process. Intergroup Relat.* 22 98–115. 10.1177/1368430217706741

[B85] Griffin S. (1971). Rape: the all-American crime. *Ramparts* 10 26–35.

[B86] Grubb A. Harrower J. (2008). Attribution of blame in cases of rape: an analysis of participant gender, type of rape and perceived similarity to the victim. *Aggress. Violent Behav.* 13 396–405. 10.1016/j.avb.2008.06.006

[B87] Grubb A. Turner E. (2012). Attribution of blame in rape cases: a review of the impact of rape myth acceptance, gender role conformity and substance use on victim blaming. *Aggress. Violent Behav.* 17 443–452. 10.1016/j.avb.2012.06.002

[B88] Hafer C. L. (2000). Do innocent victims threaten the belief in a just world? Evidence from a modified stroop task. *J. Pers. Soc. Psychol.* 79 165–173. 10.1037/0022-3514.79.2.165 10948971

[B89] Hammock G. S. Richardson D. R. (1997). Perceptions of rape: the influence of closeness of relationship, intoxication and sex of participant. *Violence Vict.* 12 237–246. 10.1891/0886-6708.12.3.237 9477539

[B90] Hammond E. M. Berry M. A. Rodriquez D. N. (2011). The influence of rape myth acceptance, sexual attitudes, and belief in a just world on attributions of responsibility in a date rape scenario. *Legal Criminol. Psychol.* 16 242–252. 10.1348/135532510X49987

[B91] Harbottle S. (2015). *Predictor Variables for Blame of Victims of Sexual Assault (Order No. AAI3581498).* Available at: http://search.proquest.com/docview/1689320148?accountid=14556

[B92] Harned M. S. (2004). Does It Matter What You Call It? The relationship between labeling unwanted sexual experiences and distress. *J. Consult. Clin. Psychol.* 72 1090–1099. 10.1037/0022-006X.72.6.1090 15612855

[B93] Hayes R. M. Lorenz K. Bell K. A. (2013). Victim blaming others: rape myth acceptance and the just world belief. *Fem. Criminol.* 8 202–220. 10.1177/1557085113484788

[B94] Hayes-Smith R. M. Levett L. M. (2010). Student perceptions of sexual assault resources and prevalence of rape myth attitudes. *Fem. Criminol.* 5 335–354. 10.1177/1557085110387581

[B95] Hill R. (1972). *The Strengths of Black Families.* New York, NY: Emerson-Hall.

[B96] Hinch S. S. Thomas R. W. (1999). Rape myth acceptance and college students: how far we have come. *Sex Roles* 40 815–832. 10.1023/A:1018816920168

[B97] Ho C. K. (1990). An analysis of domestic violence in Asian American communities: a multicultural approach to counseling. *Women Ther.* 9 129–150. 10.1300/J015v09n01_08

[B99] Hoffman H. P. Miller A. S. (1997). Social and political attitudes among religious groups: convergence and Divergence over time. *J. Sci. Study Relig.* 36 52–70. 10.2307/1387882

[B98] hooks b. (1994). *Sexism and Misogyny: Who Takes the Rap? Misogyny, Gangsta Rap, and the Piano.* Hull, MA: Z Magazine.

[B100] Howells K. Shaw E. Greasley M. Robertson J. Gloster D. Metcalfe N. (1984). Perceptions of rape in a British sample: effects of relationship, victim status, sex, and attitudes to women. *Br. J. Soc. Psychol.* 23 35–40. 10.1111/j.2044-8309.1984.tb00606.x 6697079

[B101] Idsis Y. Edoute A. (2017). Attribution of blame to rape victims and offenders, and attribution of severity in rape cases: non-therapists and survivor and offender therapists. *Int. Rev. Victimol.* 23 257–274. 10.1177/0269758017711980

[B102] Janoff-Bulman R. Timko C. Carli L. L. (1985). Cognitive biases in blaming the victim. *J. Exp. Soc. Psychol.* 21 161–177. 10.1016/0022-1031(85)90013-7

[B103] Jimenez J. A. (2002). *The Effects of Race and Gender on Respondent Attitudinal Perceptions of Acquaintance Rape (Order No. AAI3041473).* Available at: http://search.proquest.com/docview/619952494?accountid=14556

[B104] Jimenez J. A. Abreu J. M. (2003). Race and sex effects on attitudinal perceptions of acquaintance rape. *J. Couns. Psychol.* 50 252–256. 10.1037/0022-0167.50.2.252

[B105] Johnson J. D. (1994). The effect of rape type and information admissibility on perceptions of rape victims. *Sex Roles* 30 781–792. 10.1007/BF01544231

[B106] Johnson J. D. Jackson L. A. Jr. (1988). Assessing the effects of factors that might underlie the differential perception of acquaintance and stranger rape. *Sex Roles* 19 37–45. 10.1007/BF00292462

[B107] Johnson J. D. Jackson L. A. Gatto L. Nowak A. (1995). Differential male and female responses to inadmissible sexual history information regarding a rape victim. *Basic Appl. Soc. Psychol.* 16 503–513. 10.1207/s15324834basp1604_7

[B108] Johnson J. D. Jackson L. A. Smith G. J. (1989). The role of ambiguity and gender in mediating the effects of salient cognitions. *Pers. Soc. Psychol. Bull.* 15 52–60. 10.1177/0146167289151005

[B109] Johnson K. K. P. (1995). Attributions about date rape: impact of clothing, sex, money spent, date type, and perceived similarity. *Fam. Consum. Sci. Res. J.* 23 292–310. 10.1177/1077727X95233004

[B110] Johnson K. P. Ju H. W. Wu J. (2016). Young adults’ inferences surrounding an alleged sexual assault: alcohol consumption, gender, dress, and appearance schematicity. *Cloth. Textiles Res. J.* 34 127–142. 10.1177/0887302X15624550

[B111] Jones C. Aronson E. (1973). Attribution of fault to a rape victim as a function of respectability of the victim. *J. Pers. Soc. Psychol.* 26 415–419. 10.1037/h0034463 4710112

[B112] Kahn A. Gilbert L. A. Latta R. M. Deutsch C. Hagen R. Hill M. (1977). Attribution of fault to a rape victim as a function of respectability of the victim: a failure to replicate or extend. *Represent. Res. Soc. Psychol.* 8 98–107.

[B113] Kalof L. (1999). The effects of gender and music video imagery on sexual attitudes. *J. Soc. Psychol.* 139 378–385. 10.1080/00224549909598393 10410622

[B114] Kanekar S. Nazareth A. M. (1988). Attributed rape victim’s fault as a function of her attractiveness, physical hurt, and emotional disturbance. *Soc. Behav.* 3 37–40.

[B115] Kanekar S. Seksaria V. (1993). Acquaintance versus stranger rape: testing the ambiguity reduction hypothesis. *Eur. J. Soc. Psychol.* 23 485–494. 10.1002/ejsp.2420230506

[B116] Kanekar S. Shaherwalla A. Franco B. Kunju T. Pinto A. J. (1991). The acquaintance predicament of a rape victim. *J. Appl. Soc. Psychol.* 21 1524–1544. 10.1111/j.1559-1816.1991.tb00486.x

[B117] Katz I. Hass R. G. (1988). Racial ambivalence and American value conflict: correlational and priming studies of dual cognitive structures. *J. Pers. Soc. Psychol.* 55 893–905. 10.1037/0022-3514.55.6.893

[B118] Kerr N. L. Kurtz S. T. (1977). Effects of a victim’s suffering and respectability on mock juror judgments: further evidence for the just world theory. *Represent. Res. Soc. Psychol.* 8 42–56.

[B119] Kilpatrick D. G. Resnick H. S. Ruggiero K. J. Conoscenti L. M. McCauley J. (2007). *Drug Facilitated, Incapacitated, and Forcible Rape: A National Study (NCJ 219181).* Charleston, SC: Medical University of South Carolina.

[B120] Kleinke C. L. Meyer C. (1990). Evaluation of rape victims by men and women with high and low belief in a just world. *Psychol. Women Q.* 14 343–353. 10.1111/j.1471-6402.1990.tb00024.x

[B121] Klippenstine M. A. Schuller R. A. Wall A. (2007). Perceptions of sexual assault: the expression of gender differences and the impact of target alcohol consumption. *J. Appl. Soc. Psychol.* 37 2620–2641. 10.1111/j.1559-1816.2007.00273.x

[B122] Kopper B. A. (1996). Gender, gender identity, rape myth acceptance, and time of initial resistance on the perception of acquaintance rape blame and avoidability. *Sex Roles* 34 81–93. 10.1007/BF01544797

[B123] Koss M. P. Dinero T. E. Seibel C. A. Cox S. L. (1988). Stranger and acquaintance rape: are there differences in the victim’s experience? *Psychol. Women Q.* 12 1–24. 10.1111/j.1471-6402.1988.tb00924.x

[B124] Koss M. P. Gaines J. A. (1993). The prediction of sexual aggression by alcohol use, athletic participation, and fraternity affiliation. *J. Interpers. Violence* 8 94–108. 10.1177/088626093008001007

[B125] Koss M. P. Gidycz C. A. Wisniewski N. (1987). The scope of rape: incidence and prevalence of sexual aggression and victimization in a national sample of higher education students. *J. Consult. Clin. Psychol.* 55 162–170. 10.1037//0022-006X.55.2.162 3494755

[B126] Koss M. P. Harvey M. R. (1991). *The Rape Victim: Clinical and Community Interventions*, 2nd Edn Thousand Oaks, CA: Sage Publications.

[B127] Koss M. P. Heise L. Russo N. F. (1994). The global health burden of rape. *Psychol. Women Q.* 18 509–537. 10.1111/j.1471-6402.1994.tb01046.x

[B128] Krahé B. (1988). Victim and observer characteristics as determinants of responsibility attributions to victims of rape. *J. Appl. Soc. Psychol.* 18 50–58. 10.1111/j.1559-1816.1988.tb00004.x

[B129] Krahé B. Temkin J. Bieneck S. (2007). Schema-driven information processing in judgements about rape. *Appl. Cogn. Psychol.* 21 602–619. 10.1002/acp.1297

[B130] Krebs C. P. Lindquist C. H. Warner T. D. Fisher B. S. Martin S. L. (2009). College women’s experiences with physically forced, alcohol or other drug-enabled, and drug-facilitated sexual assault before and since entering college. *J. Am. Coll. Health* 57 639–647. 10.3200/JACH.57.6.639-649 19433402

[B131] Krebs D. (1975). Empathy and altruism. *J. Pers. Soc. Psychol.* 32 1134–1146. 10.1037/0022-3514.32.6.1134 1214217

[B132] Krulewitz J. E. Nash J. E. (1979). Effects of rape victim resistance, assault outcome, and sex of observer on attributions of rape. *J. Pers.* 47 557–574. 10.1111/j.1467-6494.1979.tb00209.x 533862

[B133] Lambert A. J. Raichle K. (2000). The role of political ideology in mediating judgments of blame in rape victims and their assailants: a test of the just world, personal responsibility and legitimization hypotheses. *Pers. Soc. Psychol. Bull.* 26 853–863. 10.1177/0146167200269010

[B134] Landström S. Strömwall L. A. Alfredsson H. (2016). Blame attributions in sexual crimes: effects of belief in a just world and victim behavior. *Nord. Psychol.* 68 2–11. 10.1080/19012276.2015.1026921

[B135] Langley T. Yost E. A. O’Neal E. C. Taylor S. L. Frankel P. I. Craig K. M. (1991). Models of rape judgment: attributions concerning event, perpetrator, and victim. *J. Offender Rehabil.* 17 43–54. 10.1300/J076v17n01_04

[B136] Lanis K. Covell K. (1995). Images of women in advertisements: effects on attitudes related to sexual aggression. *Sex Roles* 32 639–649. 10.1007/BF01544216

[B137] Larcombe W. (2002). The ‘ideal’ victim v successful rape complainants: not what you might expect. *Fem. Legal Stud.* 10 131–148. 10.1023/A:1016060424945

[B138] L’Armand K. Pepitone A. (1982). Judgments of rape: a study of victim–rapist relationship and victim sexual history. *Pers. Soc. Psychol. Bull.* 8 134–139. 10.1177/014616728281021

[B139] Lerner M. J. (1970). “The desire for justice and reactions to victims,” in *Altruism and Helping Behavior*, eds Macaulay J. Berkowitz L. (New York, NY: Academic Press).

[B140] Lerner M. J. (1980). *The Belief in a Just World: A Fundamental Delusion.* New York, NY: Springer 9–30. 10.1007/978-1-4899-0448-5_2

[B141] Lerner M. J. Miller D. T. (1978). Just world research and the attribution process: looking back and ahead. *Psychol. Bull.* 85 1030–1051. 10.1037/0033-2909.85.5.1030

[B142] Locke B. D. Mahalik J. R. (2005). Examining masculinity norms, problem drinking, and athletic involvement as predictors of sexual aggression in college men. *J. Couns. Psychol.* 52 279–283. 10.1037/0022-0167.52.3.279

[B143] Longino H. (1980). “Pornography, oppression and freedom: a closer look,” in *Take Back the Night*, ed. Lederer L. (New York, NY: William Morrow Co).

[B144] Lonsway K. A. Fitzgerald L. F. (1994). Rape myths: in review. *Psychol. Women Q.* 18 133–164. 10.1111/j.1471-6402.1994.tb00448.x

[B145] Loughnan S. Pina A. Vasquez E. A. Puvia E. (2013). Sexual objectification increases rape victim blame and decreases perceived suffering. *Psychol. Women Q.* 37 455–461. 10.1177/0361684313485718

[B146] MacKinnon C. A. (1985). Pornography, civil rights, and speech. *Harv. CR-CLL Rev.* 20 1.

[B147] MacKay N. J. Covell K. (1997). The impact of women in advertisements on attitudes toward women. *Sex Roles* 36 573–583. 10.1023/A:1025613923786

[B148] Malamuth N. M. Check J. V. (1981). The effects of mass media exposure on acceptance of violence against women: a field experiment. *J. Res. Pers.* 15 436–446. 10.1016/0092-6566(81)90040-4

[B149] Margolin L. Miller M. Moran P. B. (1989). When a kiss is not just a kiss: relating violations of consent in kissing to rape myth acceptance. *Sex Roles* 20 231–243. 10.1007/BF00287721

[B150] Masser B. Lee K. McKimmie B. M. (2010). Bad woman, bad victim? Disentangling the effects of victim stereotypicality, gender stereotypicality and benevolent sexism on acquaintance rape victim blame. *Sex Roles* 62 494–504. 10.1007/s11199-009-9648-y

[B151] Maurer T. W. Robinson D. W. (2008). Effects of attire, alcohol, and gender on perceptions of date rape. *Sex Roles* 58 423–434. 10.1007/s11199-007-9343-9

[B152] McCaul K. D. Veltum L. G. Boyechko V. Crawford J. J. (1990). Understanding attributions of victim blame for rape: sex, violence, and foreseeability. *J. Appl. Soc. Psychol.* 20 1–26. 10.1111/j.1559-1816.1990.tb00375.x

[B153] McCray K. L. (2014). Intercollegiate athletes and sexual violence: a review of literature and recommendations for future study. *Trauma Violence Abuse* 16 438–443. 10.1177/1524838014537907 24903398

[B154] McKimmie B. M. Masser B. M. Bongiorno R. (2014). What counts as rape? The effect of offense prototypes, victim stereotypes, and participant gender on how the complainant and defendant are perceived. *J. Interpers. Violence* 9 2273–2303. 10.1177/0886260513518843 24470567

[B155] Melnick M. (1992). Male athletes and sexual assault. *J. Phys. Ther. Educ. Recreation Dance* 63 32–36. 10.1080/07303084.1992.10604186

[B156] Miller A. K. Markman K. D. Amacker A. M. Menaker T. A. (2012). Expressed sexual assault legal context and victim culpability attributions. *J. Interpers. Violence* 27 1023–1039. 10.1177/0886260511424493 22048875

[B157] Monson C. M. Langhinrichsen-Rohling J. Binderup T. (2000). Does ”no” really mean ”no” after you say ”yes”? Attributions about date and marital rape. *J. Interpers. Violence* 15 1156–1174. 10.1177/088626000015011003

[B158] Mori L. Bernat J. A. Glenn P. A. Selle L. L. Zarate M. G. (1995). Attitudes toward rape: gender and ethnic differences across Asian and Caucasian college students. *Sex Roles* 32 457–467. 10.1007/BF01544182

[B159] Muehlenhard C. L. Hollabaugh L. C. (1988). Do women sometimes say no when they mean yes? The prevalence and correlates of women’s token resistance to sex. *J. Pers. Soc. Psychol.* 54 872–879. 10.1037/0022-3514.54.5.872 3379584

[B160] Muehlenhard C. L. Linton M. A. (1987). Date rape and sexual aggression in dating situations: incidence and risk factors. *J. Couns. Psychol.* 34 186–196. 10.1037/0022-0167.34.2.186

[B161] Muehlenhard C. L. MacNaughton J. S. (1988). Women’s beliefs about women who “lead men on.” *J. Soc. Clin. Psychol.* 7 65–79. 10.1521/jscp.1988.7.1.65

[B162] Muehlenhard C. L. McCoy M. L. (1991). Double standard/double bind: the sexual double standard and women’s communication about sex. *Psychol. Women Q.* 15 447–461. 10.1111/j.1471-6402.1991.tb00420.x 8853967

[B163] Muehlenhard C. L. Quackenbush D. M. (1998). “Sexual double standard scale,” in *Handbook of Sexuality-Related Measures*, eds Davis C. M. Yarber W. L. Bauserman R. Schreer G. Davis S. L. (Thousand Oaks, CA: Sage), 186–188.

[B164] Munsch C. L. Willer R. (2012). The role of gender identity threat in perceptions of date rape and sexual coercion. *Violence Against Women* 18 1125–1146. 10.1177/1077801212465151 23136179

[B165] Nario-Redmond M. R. Branscombe N. R. (1996). It could have been better or it might have been worse: implications for blame assignment in rape cases. *Basic Appl. Soc. Psychol.* 18 347–366. 10.1207/s15324834basp1803_6

[B166] Ohbuchi K. Ikeda T. Takeuchi G. (1994). Effects of violent pornography upon viewers’ rape myth beliefs: a study of Japanese males. *Psychol. Crime Law* 1 71–81. 10.1080/10683169408411937

[B167] Ong A. S. J. Ward C. A. (1999). The effects of sex and power schemas, attitudes toward women, and victim resistance on rape attributions. *J. Appl. Soc. Psychol.* 29 362–376. 10.1111/j.1559-1816.1999.tb01391.x

[B168] O’Toole L. L. (2007). “Subcultural theory of rape revisited,” in *Gender Violence: Interdisciplinary Perspectives*, 2nd Edn, eds O’Toole L. L. Schiffman J. R. Edwards M. L. K. (New York, NY: New York University Press), 214–222.

[B169] Pagelow M. D. (1988). “Marital rape,” in *Handbook of Family Violence*, eds Van Hasselt V. B. Morrison R. L. Bellack A. S. Hersen M. (New York, NY: Springer), 207–232. 10.1007/978-1-4757-5360-8_9

[B170] Payne D. L. Lonsway K. A. Fitzgerald L. F. (1999). Rape myth acceptance: exploration of its structure and its measurement using the Illinois Rape Myth Acceptance Scale. *J. Res. Pers.* 33 27–68. 10.1006/jrpe.1998.2238

[B171] Pederson S. H. Strömwall L. A. (2013). Victim blame, sexism, and just-world beliefs: a cross-cultural comparison. *Psychiatry Psychol. Law* 20 932–941. 10.1080/13218719.2013.770715

[B172] Persson S. Dhingra K. Grogan S. (2018). Attributions of victim blame in stranger and acquaintance rape: a quantitative study. *J. Clin. Nurs.* 27 1–10. 10.1111/jocn.14351 29518275

[B173] Pfeiffer M. G. (1990). Date rape: the reality. *South. Univ. Law Rev.* 17 283–295. 11974442

[B174] Pollard P. (1992). Judgments about victims and attackers in depicted rapes: a review. *Br. J. Soc. Psychol.* 31 309–326. 10.1111/j.2044-8309.1992.tb00975.x 1472985

[B175] Pratto F. (1996). “Sexual politics: the gender gap in the bedroom, the cupboard, and the cabinet,” in *Sex, Power, Conflict: Evolutionary and Feminist Perspectives*, eds Buss D. M. Malamuth N. M. (New York, NY: Oxford University Press), 179–230.

[B176] Pratto F. Stallworth L. M. Sidanius J. (1997). The gender gap: differences in political attitudes and social dominance orientation. *Br. J. Soc. Psychol.* 36 49–68. 10.1111/j.2044-8309.1997.tb01118.x 9114484

[B177] Pugh M. D. (1983). Contributory fault and rape convictions: loglinear models for blaming the victim. *Soc. Psychol. Q.* 46 233–242. 10.2307/3033794 6635697

[B178] Qi S. J. Starfelt L. C. White K. M. (2016). Attributions of responsibility, blame and justifiability to a perpetrator and victim in an acquaintance rape scenario: the influence of Marijuana intoxication. *J. Sex. Aggress.* 22 20–35. 10.1080/13552600.2015.1025868

[B179] Quackenbush R. L. (1989). A comparison of androgynous, masculine sex-typed and undifferentiated males on dimensions of attitudes toward rape. *J. Res. Pers.* 23 318–342. 10.1016/0092-6566(89)90005-6

[B180] Rennison D. M. (2002). *Rape and Sexual Assault: Reporting to Police and Medical Attention, 1992-2000.* Washington, DC: U.S. Department of Justice.

[B181] Richardson D. C. Campbell L. (1980). Alcohol and wife abuse: the effect of alcohol on attributions of blame for wife abuse. *Pers. Soc. Psychol. Bull.* 6 51–56. 10.1177/014616728061007

[B182] Richardson D. C. Campbell L. (1982). Alcohol and rape: the effect of alcohol on attributions of blame for rape. *Pers. Soc. Psychol. Bull.* 8 468–476. 10.1177/0146167282083013

[B183] Richardson D. R. Hammock G. S. (1991). “Alcohol and acquaintance rape,” in *Acquaintance Rape: The Hidden Crime*, eds Parrot A. Bechofer L. (New York, NY: Wiley), 83–95.

[B184] Romero-Sánchez M. Megías J. L. Krahé B. (2012). The role of alcohol and victim sexual interest in Spanish students’ perceptions of sexual assault. *J. Interpers. Violence* 27 2230–2258. 10.1177/0886260511432149 22203631

[B185] Root L. P. A. (1993). Reactions to stranger and acquaintance rape: a study of causal attributions and behavioral intentions toward victims (University of Mississippi). *Diss. Abstr. Int.* 48:2106B.

[B186] Rozée P. D. (1993). Forbidden or forgiven? Rape in cross-cultural perspective. *Psychol. Women Q.* 17 499–514. 10.1111/j.1471-6402.1993.tb00658.x

[B187] Rubin Z. Peplau A. (1973). Belief in a just world and reactions to another’s lot: a study of participants in the national draft lottery. *J. Soc. Issues* 29 73–93. 10.1111/j.1540-4560.1973.tb00104.x

[B188] Ruggi S. (1998). Commodifying honor in female sexuality: honor killings in palestine. *Middle East Rep.* 206 12–15. 10.2307/3012473

[B189] Russell D. E. H. (1984). *Sexual Exploitation: Rape, Child Sexual Abuse, and Workplace Harassment.* Beverly Hills, CA: Sage.

[B190] Ryan W. (1971). *Blaming the Victim.* New York, NY: Pantheon.

[B192] Sanchez-Hucles J. Dutton M. A. (1999). “The interaction between societal violence and domestic violence: racial and cultural factors,” in *What Causes Men’s Violence Against Women?*, eds Harway M. O’Neil J. M. (Thousand Oaks, CA: Sage Publications), 183–203. 10.4135/9781452231921.n11

[B193] Sanday P. R. (1981). The socio-cultural context of rape: a cross-cultural study. *J. Soc. Issues* 37 5–27. 10.1111/j.1540-4560.1981.tb01068.x 21113425

[B191] Sanday P. R. (1990). *Fraternity Gang Rape: Sex, Brotherhood, and Privilege on Campus.* New York, NY: New York University Press.

[B195] Schuller R. A. McKimmie B. M. Masser B. M. Klipenstine M. A. (2010). Judgements of sexual assault: the impact of complainant emotional demeanor, gender, and victim stereotypes. *New Crim. Law Rev.* 13 759–780. 10.1525/nclr.2010.13.4.759

[B196] Schuller R. A. Wall A. (1998). The effects of defendant and complainant intoxication on mock jurors’ judgements of sexual assault. *Psychol. Women Q.* 22 555–573. 10.1111/j.1471-6402.1998.tb00177.x

[B197] Schur E. M. (1983). *Labeling Women Deviant: Gender, Stigma, and Social Control.* Philadelphia, PA: Temple University Press.

[B194] Schur E. (1988). *The Americanization of Sex.* Philadelphia, PA: Temple University Press.

[B198] Scronce C. A. Corcoran K. J. (1995). The influence of the victim’s consumption of alcohol on perceptions of stranger and acquaintance rape. *Violence Against Women* 1 241–253. 10.1177/1077801295001003004 12322334

[B199] Selby J. W. Calhoun L. G. Brock T. A. (1977). Sex differences in the social perception of rape victims. *Pers. Soc. Psychol. Bull.* 3 412–415. 10.1177/014616727700300310

[B200] Seligman C. Paschall N. Takata G. (1974). Effects of physical attractiveness on attribution of responsibility. *Can. J. Behav. Sci.* 6 290–296. 10.1037/h0081875

[B201] Shaver K. G. Drown D. (1986). On causality, responsibility, and self-blame: a theoretical note. *J. Pers. Soc. Psychol.* 50 697–702. 10.1037/0022-3514.50.4.697 3712221

[B202] Shotland R. L. Goodstein L. (1983). Just because she doesn’t want to doesn’t mean it’s rape: an experimentally based causal model of the perception of rape in a dating situation. *Soc. Psychol. Q.* 46 220–232. 10.2307/3033793 6635696

[B203] Shultz S. K. Scherman A. Marshall L. J. (2000). Evaluation of a university-based date rape prevention program: effect on attitudes and behavior related to rape. *J. Coll. Stud. Dev.* 41 193–201.

[B204] Sidanius J. Pratto F. Bobo L. (1996). Racism, conservatism, affirmative action, and intellectual sophistication: a matter of principled conservatism or group dominance? *J. Pers. Soc. Psychol.* 70 476–490. 10.1037/0022-3514.70.3.476

[B205] Simonson K. Subich L. M. (1999). Rape perceptions as a function of gender-role traditionality and victim-perpetrator association. *Sex Roles* 40 617–634. 10.1023/A:1018844231555

[B206] Sims C. M. Noel N. E. Maisto S. A. (2007). Rape blame as a function of alcohol presence and resistance type. *Addict. Behav.* 32 2766–2775. 10.1016/j.addbeh.2007.04.013 17532576

[B207] Sinclair H. C. Bourne L. E. Jr. (1998). Cycle of blame or just world: effects of legal verdicts on gender patterns in rape-myth acceptance and victim empathy. *Psychol. Women Q.* 22 575–588. 10.1111/j.1471-6402.1998.tb00178.x

[B208] Smith D. D. (1976). The social content of pornography. *J. Commun.* 26 16–24. 10.1111/j.1460-2466.1976.tb01351.x

[B209] Smith R. E. Keating J. P. Hester R. K. Mitchell E. M. (1976). Role and justice considerations in the attribution of responsibility to a rape victim. *J. Res. Pers.* 10 346–357. 10.1016/0092-6566(76)90024-6

[B210] Sommer S. Reynolds J. J. Kehn A. (2016). Mock juror perceptions of rape victims: impact of case characteristics and individual differences. *J. Interpers. Violence* 31 2847–2866. 10.1177/0886260515581907 25900913

[B211] Soothill K. (1991). The changing face of rape? *Br. J. Criminol.* 31 383–392. 10.1093/oxfordjournals.bjc.a048136

[B212] Sorenson S. B. (1996). Violence against women: examining ethnic differences and commonalities. *Eval. Rev.* 20 123–145. 10.1177/0193841X9602000201 10182200

[B213] Spencer B. (2016). The impact of class and sexuality-based stereotyping on rape blame. *Sexualization Media Soc.* 2 1–8. 10.1177/2374623816643282

[B214] Sprecher S. McKinney K. Orbuch T. L. (1987). Has the double standard disappeared? An experimental test. *Soc. Psychol. Q.* 50 24–31. 10.2307/2786887 16390354

[B215] Stahl T. Eek D. Kazemi A. (2010). Rape victim blaming as system justification: the role of gender and activation of complementary stereotypes. *Soc. Justice Res.* 23 239–258. 10.1007/s11211-010-0117-0

[B216] Stankiewicz J. M. Rosselli F. (2008). Women as sex objects and victims in print advertisements. *Sex Roles* 58 579–589. 10.1007/s11199-007-9359-1

[B217] Starfelt L. C. Young R. M. White K. M. Palk G. M. (2015). Explicating the role of sexual coercion and vulnerability alcohol expectancies in rape attributions. *J. Interpers. Violence* 30 1965–1981. 10.1177/0886260514549466 25228594

[B218] Stephan C. Tully J. C. (1977). The influence of physical attractiveness on a plaintiff on the decisions of simulated jurors. *J. Soc. Psychol.* 101 149–150. 10.1080/00224545.1977.9923997

[B219] Stormo K. J. Lang A. R. Stritzke W. G. K. (1997). Attributions about acquaintance rape: the role of alcohol and individual differences. *J. Appl. Soc. Psychol.* 27 279–305. 10.1111/j.1559-1816.1997.tb00633.x

[B220] Strömwall L. A. Alfredson H. Landstrom S. (2013). Blame attributions and rape: effects of belief in a just world and relationship level. *Legal Criminol. Psychol.* 18 254–261. 10.1111/j.2044-8333.2012.02044.x

[B221] Stuart S. M. McKimmie B. M. Masser B. M. (2016). Rape perpetrators on trial: the effect of sexual assault-related schemas on attributions of blame. *J. Interpers. Violence* 34 310–336. 10.1177/0886260516640777 27026408

[B222] Suarez E. Gadalla T. M. (2010). Stop blaming the victim: a meta-analysis on rape myths. *J. Interpers. Violence* 25 2010–2035. 10.1177/0886260509354503 20065313

[B223] Tetreault P. A. Barnett M. A. (1987). Reactions to stranger and acquaintance rape. *Psychol. Women Q.* 11 353–358. 10.1111/j.1471-6402.1987.tb00909.x

[B224] Thornton B. (1984). Defensive attribution of responsibility: evidence for an arousal-based motivational bias. *J. Pers. Soc. Psychol.* 46 721–734. 10.1037/0022-3514.46.4.721

[B225] Ullman S. E. Karabatsos G. Koss M. P. (1999). Alcohol and sexual assault in a national sample of college women. *J. Interpers. Violence* 14 603–625. 10.1177/088626099014006003

[B226] Ulman S. E. (1996). Social reactions, coping strategies, and self-blame attributions in adjustment to sexual assault. *Psychol. Women Q.* 20 505–526. 10.1111/j.1471-6402.1996.tb00319.x

[B227] Van Den Bos K. Maas M. (2009). On the psychology of the belief in a just world: exploring experiential and rationalistic path to victim blaming. *Pers. Soc. Psychol. Bull.* 35 1567–1578. 10.1177/0146167209344628 19726809

[B228] Vandello J. A. Bosson J. K. (2013). Hard won and easily lost: a review and synthesis of theory and research on precarious manhood. *Psychol. Men Masc.* 14 101–113. 10.1037/a0029826

[B229] Vandello J. A. Bosson J. K. Cohen D. Burnaford R. M. Weaver J. R. (2008). Precarious manhood. *J. Pers. Soc. Psychol.* 95 1325–1339. 10.1037/a0012453 19025286

[B230] Varelas N. Foley L. A. (1998). Blacks’ and Whites’ perceptions of interracial an intraracial date rape. *J. Soc. Psychol.* 138 392–400. 10.1080/00224549809600391 9577729

[B231] Viki G. T. Abrams D. (2002). But she was unfaithful: benevolent sexism and reactions to rape victims who violate traditional gender role expectations. *Sex Roles* 47 289–293. 10.1023/A:1021342912248

[B232] Wall A. Schuller R. A. (2002). Sexual assault and defendant/victim intoxication: jurors’ perceptions of guilt. *J. Appl. Psychol.* 30 253–274. 10.1111/j.1559-1816.2000.tb02315.x

[B233] Ward C. (1988). The attitudes toward rape victims scale: construction, validation, and cross-cultural applicability. *Psychol. Women Q.* 12 127–146. 10.1111/j.1471-6402.1988.tb00932.x

[B234] Ward C. (1995). *Attitudes Toward Rape: Feminist and Social Psychological Perspectives.* London: Sage.

[B235] Warshaw R. (1994). *I Never Called it Rape*, 2nd Edn New York, NY: Harper & Row.

[B236] Weis K. Borges S. S. (1973). Victimology and rape: the case of the legitimate victim. *Issues Criminol.* 8 71–115.

[B237] Whatley M. A. (1996). Victim characteristics influencing attributions of responsibility to rape victims: a meta-analysis. *Aggress. Violent Behav.* 1 81–95. 10.1016/1359-1789(95)00011-9

[B238] Whatley M. A. (2005). The effect of participant sex, victim dress, and traditional attitudes on causal judgments for marital rape victims. *J. Fam. Violence* 20 191–200. 10.1007/s10896-005-3655-8

[B239] Whatley M. A. Riggio R. E. (1992). Attributions of blame for female and male victims. *Fam. Violence Sex. Assault Bull.* 8 16–18.

[B240] White A. M. Potgieter C. A. Strube M. J. Fisher S. Umana E. (1997). An African-centered, Black feminist approach to understanding attitudes that counter social dominance. *J. Black Psychol.* 23 398–420. 10.1177/00957984970234007

[B242] Wiener R. L. Vodanovich S. J. (1986). The evaluation of culpability for rape: a model of legal decision making. *J. Psychol.* 120 489–500. 10.1080/00223980.1986.9915481

[B243] Williams J. E. (1984). Secondary victimization: confronting public attitudes about rape. *Victimology* 9 66–81.

[B244] Williams J. E. Holmes K. A. (1981). *The Second Assault: Rape and Public Attitudes.* Westport, CT: Greenwood Press.

[B245] Willis C. E. (1992). The effect of sex role stereotype, victim and defendant race, and prior relationship on rape culpability attributions. *Sex Roles* 26 213–226. 10.1007/BF00289708

[B246] Wooten J. N. (1980). The effects of victim/assailant familiarity and victim resistance on attitudes toward rape among law enforcement personnel and college students (Texas A & M University). *Diss. Abstr. Int.* 41:1487B.

[B247] Workman J. E. Orr R. L. (1996). Clothing, sex of subject, and rape myth acceptance as factors affecting attributions about an incident of acquaintance rape. *Cloth. Textiles Res. J.* 14 276–284. 10.1177/0887302X9601400407

[B248] World Economic Forum (2017). *The Global Gender Gap Report.* Cologny: World Economic Forum.

[B241] World Health Organization [WHO] (2005). *Multi-Country Study on Women’s Health and Domestic Violence Against Women: Summary Report of Initial Results on Prevalence, Health Outcomes and Women’s Responses.* Geneva: World Health Organization.

[B249] Wyer R. S. Jr. Bodenhausen G. V. Gorman T. F. (1985). Cognitive mediators of reactions to rape. *J. Pers. Soc. Psychol.* 48 324–338. 10.1037/0022-3514.48.2.324 3981398

[B250] Yamawaki N. (2007). Rape perception and the function of ambivalent sexism and gender-role traditionality. *J. Interpers. Violence* 22 406–422. 10.1177/0886260506297210 17369444

[B251] Yamawaki N. Darby R. Queiroz A. (2007). The moderating role of ambivalent sexism: the influence of power status on perception of rape victim and rapist. *J. Soc. Psychol.* 147 41–56. 10.3200/SOCP.147.1.41-56 17345921

[B252] Yamawaki N. Tschanz B. T. (2005). Rape perception differences between Japanese and American college students: on the mediating influence of gender-role traditionality. *Sex Roles* 52 379–392. 10.1007/s11199-005-2680-7

[B253] Young C. (1986). “Afro-American family: contemporary issues and implications for social policy,” in *On Being Black: An in-Group Analysis*, ed. Pilgrim D. (Bristol, IN: Wyndham Hall), 58–75.

[B254] Zilbergeld B. (1978). *Male Sexuality: A Guide to Sexual Fulfillment.* New York, NY: Bantam Books.

